# Current Understanding of Taxonomy and Ecology of the Phylum *Minisyncoccota*

**DOI:** 10.1264/jsme2.ME25084

**Published:** 2026-05-12

**Authors:** Naoki Fujii, Meri Nakajima, Takashi Narihiro, Kyohei Kuroda, Tomonori Kindaichi

**Affiliations:** 1 Department of Civil and Environmental Engineering, Graduate School of Advanced Science and Engineering, Hiroshima University, 1–4–1, Kagamiyama, Higashihiroshima, Hiroshima 739–8527, Japan; 2 Biomanufacturing Process Research Center, National Institute of Advanced Industrial Science and Technology (AIST), 2–17–2–1 Tsukisamu-Higashi, Toyohira-ku, Sapporo, Hokkaido 062–8517, Japan; 3 Integrated Research Center for Circular Technology, National Institute of Advanced Industrial Science and Technology (AIST), 2–17–2–1 Tsukisamu-Higashi, Toyohira-ku, Sapporo, Hokkaido, 062–8517 Japan; 4 Dairy Ecosystem Research & Development Center, Hiroshima University, 2–2965, Kagamiyama, Higashi-Hiroshima, Hiroshima, 739–0046, Japan

**Keywords:** *Minisyncoccota*, episymbiosis, genome reduction, genetic code variation, microbial interactions

## Abstract

The phylum *Minisyncoccota* (formerly known as “*Candidatus* Patescibacteria”/candidate phyla radiation [CPR] and designated under SeqCode as *Patescibacteriota*) represents one of the major bacterial phyla; however, its physiological and ecological characteristics remain unclear. This review summarizes relevant studies on currently available isolate and genomic/metagenomic data, outlining the phylogenetic placement, metabolic features, host interactions, and unique genetic code usage of *Minisyncoccota*. *Minisyncoccota* play complementary and interdependent roles within microbial communities, while being restricted by incomplete metabolic capabilities that prevent independent survival. Studies on *Minisyncoccota* offer important insights into the diversity and evolution of uncultivated bacteria, as well as the hidden interaction networks that shape microbial ecosystems.

Understanding the role played by microbial communities in diverse environments has long been constrained by reliance on the biological functions of a limited number of cultivable microorganisms. The experimental technique for directly cloning the 16S rRNA gene from environmental samples was established in the mid-1980s, laying the foundation for culture-independent molecular ecology (Pace *et al.*, 1986). This approach revealed that natural environments harbor vast microbial diversity that cannot be explained by known cultivated bacteria. Numerous “candidate phyla” were reported, highlighting the existence of broad uncultivated lineages that were not captured by traditional cultivation-based phylogenies. In recent years, advances in single-cell genomics and metagenomics have provided genomic information from uncultivated lineages present in the environment, which has led to a more detailed understanding of microbial phylogenetic classifications (Rinke *et al.*, 2013; Hug *et al.*, 2016; Parks *et al.*, 2018). *Minisyncoccota* is a prime representative of a major bacterial phylogenetic group that includes largely uncultivated lineages, for which a limited number of co-cultures and enrichment-based systems have been reported in recent years (He *et al.*, 2015; Batinovic *et al.*, 2021; Kuroda *et al.*, 2022a, 2022b, 2024; Yakimov *et al.*, 2022; Xie *et al.*, 2022; Nakajima *et al.*, 2025). Genomic anal­yses of these lineages are also progressing, and their ecological functions are becoming increasingly evident. It is important to note that‍ ‍*Minisyncoccota*, formerly known as “*Candidatus* Patescibacteria” and the candidate phyla radiation (CPR), has been published under the International Code of Nomenclature for Prokaryotes (ICNP) (Oren and Göker, 2025), while its SeqCode designation is *Patescibacteriota* (Dutkiewicz *et al.*, 2025), as of October 2025. SeqCode is a new code of nomenclature for prokaryotes that uses genome sequences as nomenclatural types (Hedlund *et al.*, 2022). Following the rejection by the International Committee on Systematics of Prokaryotes (ICSP) of proposals to incorporate DNA sequences as nomenclatural types within the ICNP, it was suggested as an independent framework to enable the naming of uncultivated microorganisms. Hereafter, unless specified otherwise, this review will use the taxonomic name *Minisyncoccota*. Several *Minisyncoccota* members have been reported in host-associated co-cultures or enrichment-based systems; however, none of these cultures were publicly available through culture collections. Although recent progress has enabled the deposition of the first two-strain co-culture of *Minisyncoccus archaeiphilus* strain PMX.108^T^ (=JCM 39522^T^) with the host archaeon, *Methanospirillum hungatei* (Nakajima *et al.*, 2025), the majority of this phylogenetic group remains uncultivated. Information on their physiology and ecology is fragmented and has yet to be systematically organized. In this review, we provide an overview of current knowledge of *Minisyncoccota*, with a focus on historical transitions in its phylogenetic classification, and summarize previous studies on PCR primer sets, fluorescence *in situ* hybridization (FISH) probes, metabolic characteristics, phylogenetic diversity, and parasitic interactions

## Taxonomic transitions and phylogenetic tree of the phylum *Minisyncoccota*

**Fig. 1.**
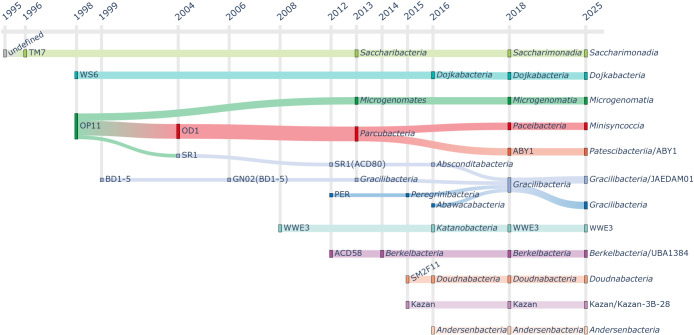
Transitions in the phylogenetic nomenclature of each class. Taxonomic names follow those originally employed in the cited literature: undefined (Bond *et al.*, 1995); TM7 (Rheims *et al.*, 1996); OP11 (Hugenholtz *et al.*, 1998); WS6 (Dojka *et al.*, 1998); BD1-5 (Li *et al.*, 1999); OD1 and SR1 (Harris *et al.*, 2004); GN02 (Ley *et al.*, 2006); WWE3 (Guermazi *et al.*, 2008); PER, ACD58, and ACD80 (Wrighton *et al.*, 2012); *Microgenomates*, *Parcubacteria*, and *Gracilibacteria* (Rinke *et al.*, 2013); *Saccharibacteria* (Albertsen *et al.*, 2013); *Berkelbacteria* (Wrighton *et al.*, 2014); *Peregrinibacteria*, Kazan, and SM2F11 (Brown *et al.*, 2015); *Absconditabacteria* and *Katanobacteria* (Hug *et al.*, 2016); *Dojkabacteria* (Wrighton *et al.*, 2016); *Abawacabacteria* and *Andersenbacteria* (Anantharaman *et al.*, 2016b); *Saccharimonadia*, *Microgenomatia*, *Paceibacteria*, and ABY1 (Parks *et al.*, 2018); *Minisyncoccia* (Nakajima *et al.*, 2025); *Patescibacteriia* (Dutkiewicz *et al.*, 2025).

*Minisyncoccota* represents a recently recognized bacterial lineage, proposed as a novel phylum with a cultured representative *Minisyncoccus archaeiphilus* within the kingdom *Bacillati* of domain *Bacteria* (Nakajima *et al.*, 2025). The taxonomic name of this group has undergone frequent revisions as new lineages have been discovered and reclassified. We herein chronologically summarized major taxonomic changes at the class level for *Minisyncoccota* reported between 1995 and the present date, tracing their historical taxonomic name in the literature and mainly aligning them with the Genome Taxonomy Database (GTDB) taxonomy Release 226 (Fig. 1) (Parks *et al.*, 2018). In this section, taxonomic names follow those originally employed in the cited literature to ensure consistency with Fig. 1. Bond *et al.* (1995) first reported partial 16S rRNA gene sequences obtained from a laboratory-scale activated sludge reactor and assigned them as “unaffiliated groups”. Rheims *et al.* (1996) proposed the designation TM7 for this unclassified lineage discovered in 16S rRNA gene clone libraries. This provisional naming convention persisted until 2013, when Rinke *et al.* introduced the taxonomy of uncultured microorganisms based on single-cell amplified genomes (SAGs) and metagenome-assembled genomes (MAGs) (Table 1). During this period, multiple novel lineages were described: OP11 from hot spring environments (Hugenholtz *et al.*, 1998), WS6 from groundwater (Dojka *et al.*, 1998), BD1-5 from deep-sea sediments (Li *et al.*, 1999), OD1 and SR1 as subdivisions of OP11 (Harris *et al.*, 2004), GN02 later recognized as being synonymous with BD1-5 (Ley *et al.*, 2006), and WWE3 from wastewater treatment facilities (Guermazi *et al.*, 2008). Miyoshi *et al.* (2005) phylogenetically characterized microorganisms captured from deep aquifers in Japan using filters with pore sizes of 0.2 and 0.1 μm. Their findings revealed that candidate divisions OD1 and OP11 were highly enriched in the filtered fraction, providing the first evidence that these lineages consist of ultra-small bacteria. Wrighton *et al.* (2012) applied metagenomics and proteomics to groundwater samples, reporting additional lineages including PER, ACD58, and ACD80. Rinke *et al.* (2013) conducted single-cell genomic anal­yses of diverse environmental samples and reconstructed a comprehensive phylogeny of *Bacteria* and *Archaea*. Their anal­yses indicated that OP11, OD1, and GN02 (BD1-5), previously regarded as distinct phyla, constitute a single higher-order lineage. At this stage, each group was still recognized as a phylum; however, the overarching clade was newly proposed as the “superphylum *Patescibacteria*”. Concurrently, OP11, OD1, and GN02 were renamed *Microgenomates*, *Parcubacteria*, and *Gracilibacteria*, respectively. During the‍ ‍same period, Albertsen *et al.* (2013) reconstructed a complete MAG from activated sludge that had previously been affiliated with TM7 and redefined it as the phylum *Saccharibacteria*. Wrighton *et al.* (2014) proposed *Berkelbacteria* for a lineage previously designated as ACD58. Brown *et al.* (2015) expanded on these findings, introducing the collective term “candidate phyla radiation (CPR)” to encompass OP11, OD1, and GN02 together with newly identified groups. This study proposed the superphyla *Parcubacteria* (OD1) and *Microgenomates* (OP11) and subdivided them into 14 and 11 phylum-level lineages, respectively. It also proposed three novel phyla (CPR1–3) and clarified the taxonomic name of PER as *Peregrinibacteria*. Additional lineages, such as Kazan and SM2F11, were also reported, but without prior documentation linking them to *Patescibacteria*.

Hug *et al.* (2016) reconstructed a large-scale phylogenetic tree encompassing all three domains of life based on 1,011 newly obtained MAGs. This study allowed us to visualize *Patescibacteria* as a broad phylogenetic group, together with the renaming of SR1 to *Absconditabacteria* and WWE3 to *Katanobacteria*. Anantharaman *et al.* (2016b) proposed 27 additional phylum-level lineages within CPR from a groundwater metagenomic anal­ysis, and Wrighton *et al.* (2016) reclassified WS6 as *Dojkabacteria*. Parks *et al.* (2018) proposed GTDB, introducing a genome-based standardized taxonomy that has since become the dominant framework for bacterial and archaeal systematics. Under GTDB, *Patescibacteria*/CPR were consolidated as a single phylum, with phylum-level groups being reassigned to the class rank. Names were further refined, with *Parcubacteria* being renamed as *Paceibacteria* and *Microgenomates* as *Microgenomatia*, and many previously proposed phyla were reorganized into orders or lower ranks. Several groups were further subdivided, *e.g.*, *Parcubacteria* was split into *Paceibacteria* and ABY1. Moreover, some names were replaced with provisional UBA codes (*e.g.*, *Berkelbacteria* to UBA1384). GTDB has since undergone frequent updates, further subdividing and reorganizing CPR lineages. *Gracilibacteria* has been split into *Gracilibacteria* and JAEDAM01, with the lineage originally being designated as‍ ‍*Gracilibacteria* by Rinke *et al.* (2013) and *Absconditabacteria* by Hug *et al.* (2016) assigned to JAEDAM01, whereas the lineages previously referred to as *Peregrinibacteria* by Wrighton *et al.* and *Abawacabacteria* by Anantharaman *et al.* (2016b) were reassigned to *Gracilibacteria*.

**Fig. 2.**
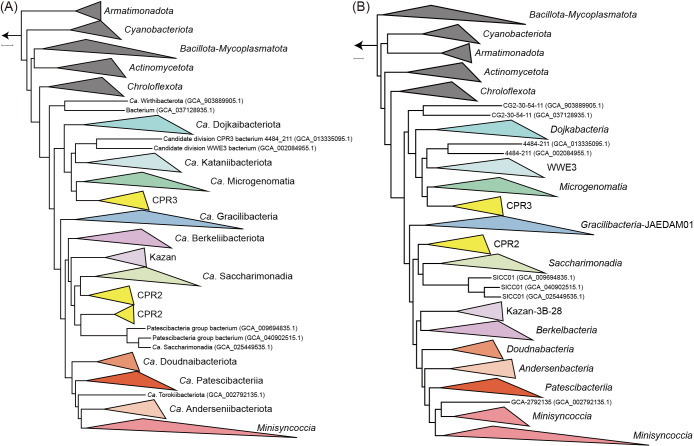
Phylogenetic tree of the phylum *Minisyncoccota*. Comparative phylogenetic anal­yses in this review, including trees reconstructed using marker proteins proposed by (A) Nakajima *et al.* (2025) and (B) the 120-marker gene set of the Genome Taxonomy DataBase (GTDB). (A) follows nomenclature consistent with the International Code of Nomenclature of Prokaryotes (ICNP), whereas (B) follows the GTDB taxonomy. Scale bars=0.1. The data used for these anal­yses are provided in Table S4.

As of 2025, two competing phylum names have been proposed for CPR. Nakajima *et al.* (2025), based on a cultivated representative, introduced *Minisyncoccota* as a novel phylum under ICNP, whereas Dutkiewicz *et al.* (2025), based on MAGs registered in SeqCode, re-proposed *Patescibacteriota* as the phylum name under SeqCode. In the List of Prokaryotic names with Standing in Nomenclature (LPSN), *Minisyncoccota* is recognized as the valid ICNP name, while *Patescibacteriota* is listed only as a *Candidatus* phylum and, thus, does not have standing under the ICNP (Freese *et al.*, 2026). GTDB currently adopts *Patescibacteriota* (Parks *et al.*, 2026) based on SeqCode. Additionally, *Paceibacteria* was renamed *Minisyncoccia* under ICNP, while ABY1 was renamed *Patescibacteriia* under SeqCode. Current phylogenetic correspondences are listed in Table 1. In summary, the taxonomic identity of the phylum *Minisyncoccota* has undergone substantial revisions since the mid-1990s. Notably, the establishment of GTDB in 2018 provided a genome-based standardized taxonomy, serving as a turning point for the systematic reclassification of *Minisyncoccota* and related lineages. Nakajima *et al.* (2025) demonstrated that *Minisyncoccota* constituted a monophyletic phylum that formed a sister relationship with *Chloroflexota*, even when different marker gene sets were used. This phylogenetic placement is also consistent with recent studies on bacterial evolution (Coleman *et al.*, 2021; Beaud *et al.*, 2025). Comparative phylogenetic anal­yses in this review, including trees reconstructed using concatenated 120 single-copy marker genes based on GTDB and a subset only including replication/transcription/translation-related genes (Nishihara *et al.*, 2024; Nakajima *et al.*, 2025) (Fig. 2), supported the monophyly of *Minisyncoccota* and its sister relationship with *Chloroflexota*. Furthermore, positional inconsistencies among certain lineages were revealed. These discrepancies, which were also observed by Nakajima *et al.* (2025) highlight the impact of marker gene selection and methodological differences on phylogenetic inference, and underscore the importance of continued refinement to resolve the precise taxonomic placement of *Minisyncoccota*.

## Detection of the phylum *Minisyncoccota*

PCR primer sets and FISH probes targeting the phylum *Minisyncoccota* have been developed for the 16S rRNA gene and, in some lineages, also for the 23S rRNA gene, enabling the specific detection of this phylum. The primers developed to date are summarized in Table S1 and S2. Amplicon sequencing using the commonly employed V4 and V3–V4 primer sets also detect *Minisyncoccota* (Hu *et al.*, 2024). However, intron insertions are frequently found in the 16S rRNA genes of *Minisyncoccota* (Brown *et al.*, 2015; Tsurumaki *et al.*, 2024), which may hinder detection in certain lineages. Hu *et al.* (2024) modified conventional V4 primers to enhance the detection rate of *Minisyncoccota* without compromising the overall diversity of the bacteria detected. Among the lineages detected, “*Ca.* Saccharimonadia” has attracted attention, and numerous PCR primers have been designed, partly due to the establishment of methods for obtaining stable co-cultures. In contrast, although “*Ca.* Patescibacteriia” and “*Ca.* Microgenomatia” are frequently identified in environmental samples, information on specific primer sets remains limited. FISH probes are fewer in number and are generally more specific than PCR primer sets, with only TM7905 (Hugenholtz *et al.*, 2001) and Pac683 (Singleton *et al.*, 2021) being designed to cover broader phylogenetic ranges. Since the PCR primers and FISH probes developed during the early stages of research on *Minisyncoccota* were based on limited reference data, their specificity needs to be confirmed when applying them to current research.

## Microorganisms forming parasitic or predatory relationships with the phylum *Minisyncoccota*

**Fig. 3.**
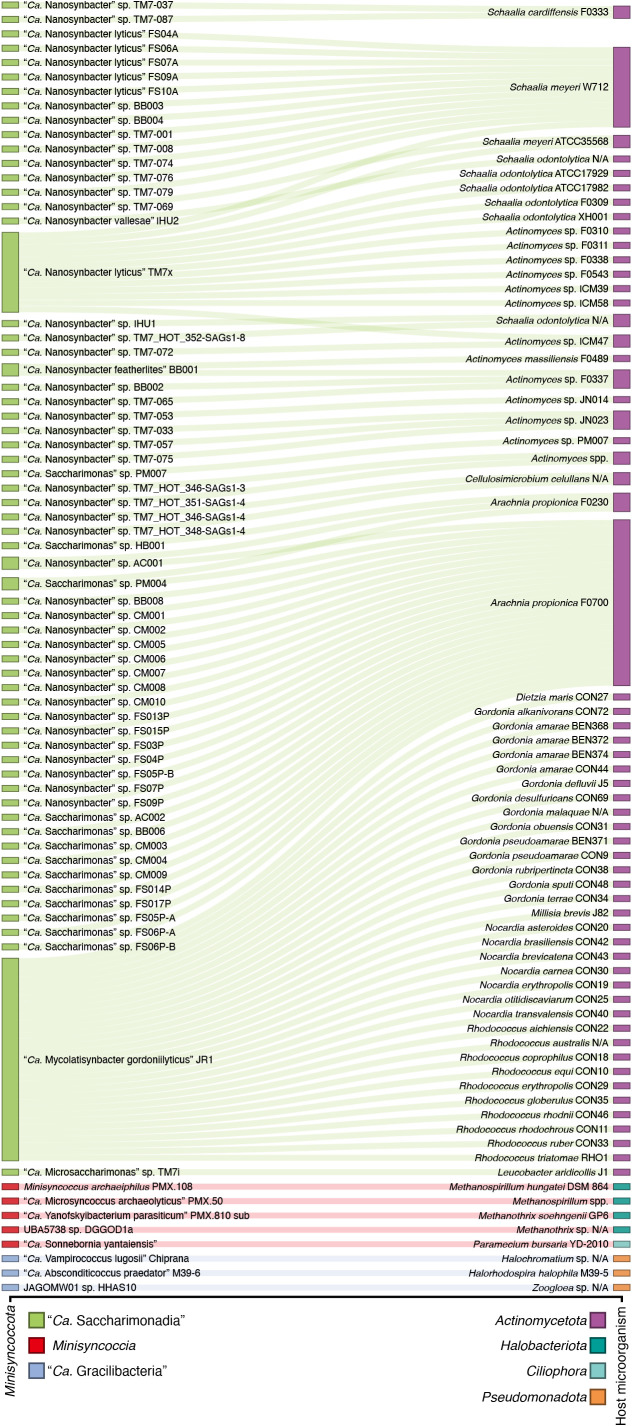
Relationships between *Minisyncoccota* strains and their host microorganisms. Species and strain names are provided. Detail information is described in Table S3.

The phylum *Minisyncoccota* is characterized by highly reduced MAG sizes and limited metabolic capabilities, suggesting that members of this lineage are generally unable to persist independently in the environment. Therefore, it is being increasingly reported that these microorganisms adopt symbiotic, parasitic, or predatory lifestyles that are dependent on other microbes (Fig. 3, Table S3). The oral environment has served as a representative model system in which “*Ca.* Nanosynbacter” species have been extensively investigated. “*Ca.* Nanosynbacter lyticus” strain TM7x was shown to associate with *Schaaria odontolytica* and *Schaaria meyeri* as host organisms, with cultivation experiments demonstrating its epibiotic lifestyle (He *et al.*, 2015; Bor *et al.*, 2016, 2018; Utter *et al.*, 2020). Furthermore, co-cultures with *Arachnia propionica* yielded multiple isolates of “*Ca.* Nanosynbacter” and “*Ca.* Saccharimonas”, in which attachment and proliferation on the host cell surface were observed (Bor *et al.*, 2020; Mukurgkar *et al.*, 2020). These findings indicate that members of “*Ca.* Saccharimonadia” preferentially use bacteria within the phylum *Actinomycetota* as primary hosts. “*Ca.* Mycolatisynbacter gordoniilyticus” (synonym “*Ca.* Mycosynbacter amalyticus”), another member of “*Ca.* Saccharimonadia”, has been shown to engage in symbiosis with *Gordonia amarae* in activated sludge (Batinovic *et al.*, 2021).

Members of “*Ca.* Gracilibacteria” (JAEDAM01 lineage in GTDB), such as “*Ca.* Vampirococcus lugosii”, “*Ca.* Absconditicoccus praedator”, and JAGOMW01-related organisms (in GTDB), have been identified as potential predators or symbionts of the class *Gammaproteobacteria*, including photosynthetic bacteria and *Zoogloea* (Moreira *et al.*, 2021; Yakimov *et al.*, 2022; Fujii *et al.*, 2024), suggesting that this lineage preferentially establishes relationships with members of this class. Several novel lineages within *Minisyncoccia* have recently been reported to parasitize archaea. “*Ca.* Yanofskyibacterium parasiticum” and “*Ca.* Nealsoniibacteriota” (synonym “*Ca.* Nealsonbacteria”) were found to proliferate in association with *Methanothrix* spp. (Kuroda *et al.*, 2022b, 2024; Chen *et al.*, 2023), while *Minisyncoccus archaeiphilus* and “*Ca.* Microsyncoccus archaeolyticus” were associated with members of the genus *Methanospirillum* (Kuroda *et al.*, 2022a, 2022b, 2024; Nakajima *et al.*, 2025). Additionally, cases of *Minisyncoccia* inhabiting eukaryotic hosts have been documented (Gong *et al.*, 2014) (Table S3). Collectively, these findings show that *Minisyncoccota* members establish parasitic or symbiotic relationships not only with bacteria, but also with archaea and eukaryotes, spanning across all three domains of life.

## Morphological characteristics

**Fig. 4.**
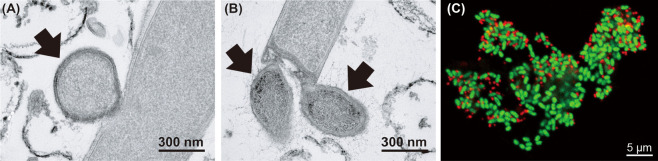
Transmission electron micrographs of (A) “*Ca.* Yanofskyibacterium parasiticum” parasitizing a *Methanothrix* cell, and (B) *Minisyncoccus archaeiphilus* parasitizing a *Methanospirillum* cell; arrows indicate *Minisyncoccota* cells (Reprinted with permission from the publisher from Kuroda *et al.*, 2023a), and (C) fluorescence *in situ* hybridization (FISH) of uncultured clade “*Ca.* Gracilibacteria”/JAEDAM01 (red) and *Zoogloea* (green). The methods used to obtain the figures are described in Kuroda *et al.* (2023a) and Fujii *et al.* (2024).

Members of the phylum *Minisyncoccota* are generally characterized by coccoid or ovoid cell morphologies, although larger cells have also been reported, suggesting morphological diversity. Cell sizes typically range from approximately 200 to 800 nm in diameter, highlighting their small dimensions (Table 2). Therefore, fractionation by filtration may be an effective approach for recovering *Minisyncoccota* cells (Bor *et al.*, 2018; Kagemasa *et al.*, 2022, 2025). The presence of an S-layer has been reported in some species (Yakimov *et al.*, 2022; Chen *et al.*, 2023). Fig. 4 shows microscopic images of *Minisyncoccota* in a wastewater treatment system. *Minisyncoccus archaeiphilus* and “*Ca.* Yanofskyibacterium parasiticum” were detected in close proximity to methanogens, playing a crucial role as an anchor for organic matter decomposition in methanogenic environments (Fig. 4A and B) (Kuroda *et al.*, 2022a, 2022b, 2023a, 2024; Nakajima *et al.*, 2025). The cells belonging to‍ ‍an uncultured lineage within “*Ca.* Gracilibacteria”/JAEDAM01 lineage often coexisted with members of the genus *Zoogloea*, known for its extracellular polymeric substance (EPS)-producing, floc-forming bacteria (Fig. 4C) (Fujii *et al.*, 2024). This close spatial association with host cells may represent one of the characteristic morphological features of *Minisyncoccota*. Previous studies demonstrated that “*Ca.* Mycolatisynbacter gordoniilyticus” attached to the cell surface of *Gordonia amarae*, a causative agent of sludge bulking, and existed in a parasitic relationship in wastewater treatment reactors (Batinovic *et al.*, 2021). In addition, oral “*Ca.* Nanosynbacter” species have been shown to parasitize host bacteria through a close physical association, thereby attenuating host pathogenicity (Chipashvili *et al.*, 2021). Taken together, these host-associated lifestyles involving direct attachment are consistent with the shared morphological traits of *Minisyncoccota* and provide important insights into their ecological strategies across different environments, as well as their potential for future applications.

## Metabolic characteristics

**Fig. 5.**
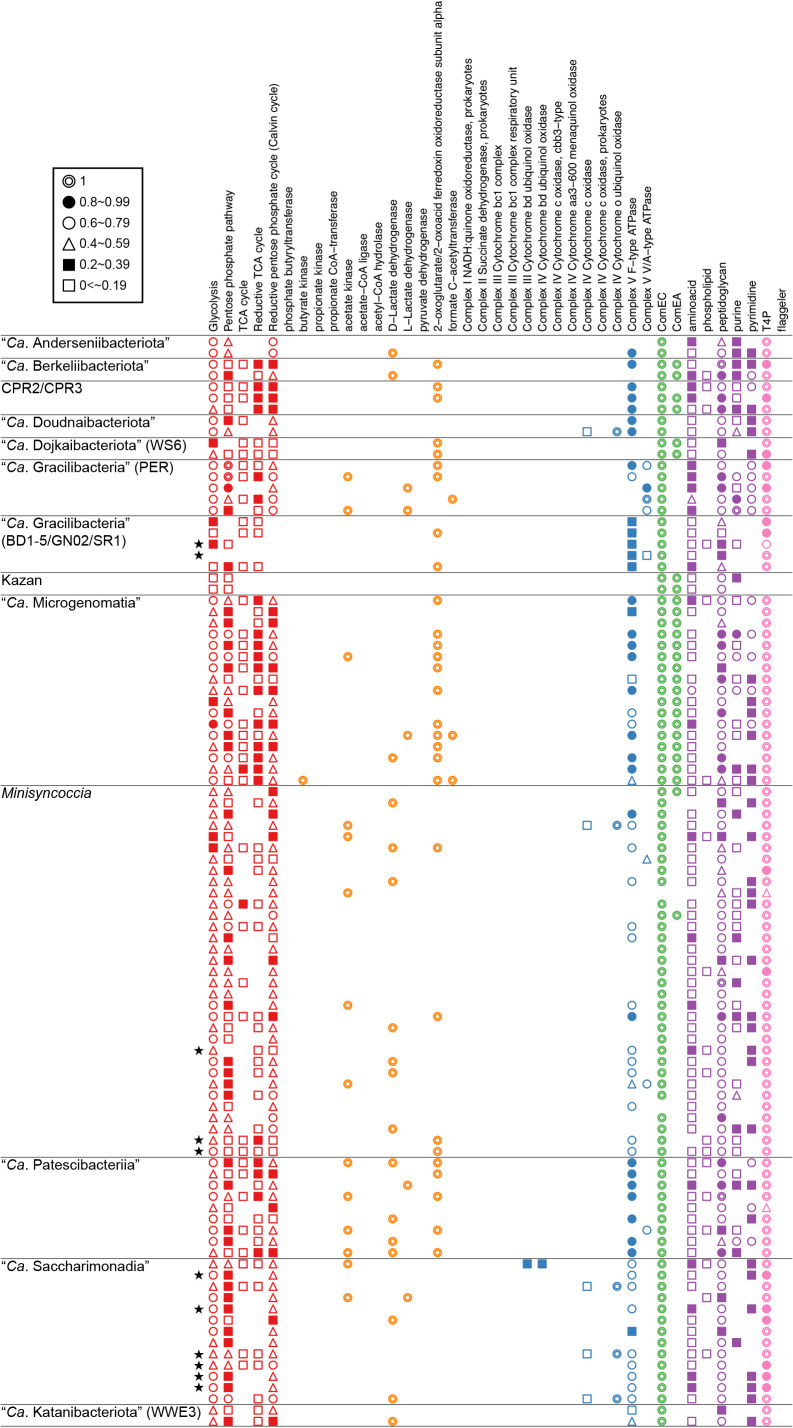
Multi-symbol annotation map of metabolic features based on the findings of Prokka v1.13.0 (Seemann, 2014), DRAM v1.5 (Shaffer *et al.*, 2020), and the MacsyFinder TFFScan or TXXScan model (Abby *et al.*, 2016). Stars represent cultured and enriched strains. The data used for these analyses are provided in Table S5–S12.

To provide an integrated overview of the metabolic characteristics of the phylum *Minisyncoccota*, we summarized genomic features inferred from MAGs derived from both environmental samples and cultivated strains in a heatmap based on previously published datasets (Fig. 5). Genome sizes averaged 0.92‍ ‍Mbp (ranging from 0.42 to 2.13‍ ‍Mbp), which is in accordance with previously reported values for members of this phylum. Overall, the predicted metabolic characteristics were consistent with those described in MAG-based studies and reviews (Castelle *et al.*, 2018; Bokhari *et al.*, 2020; Lemos *et al.*, 2020; Ji *et al.*, 2022; Maatouk *et al.*, 2023).

## Central metabolic pathways and the electron transport chain

Many MAGs partially retained genes associated with glycolysis, while complete pathways were rarely observed, suggesting a limited potential for full glucose catabolism and a dependence on external supplementation or the uptake of metabolic intermediates (Castelle *et al.*, 2018; Bokhari *et al.*, 2020; Lemos *et al.*, 2020; Ji *et al.*, 2022; Maatouk *et al.*, 2023). “*Ca.* Gracilibacteria”/JAEDAM01 lacked most of the pathway, a feature frequently reported in this lineage (Sieber *et al.*, 2019; Moreira *et al.*, 2021; Yakimov *et al.*, 2022; Kuroda *et al.*, 2023b; Fujii *et al.*, 2024). The pentose phosphate pathway was poorly represented, with most lineages possessing only a subset of enzymes. Similarly, the tricarboxylic acid (TCA) cycle and the reductive TCA cycle were largely absent, and when partially retained, their functions were limited to auxiliary roles for biosynthesis, such as cofactor generation (Wrighton *et al.*, 2012; Anantharaman *et al.*, 2016a; Danczak *et al.*, 2017; Lemos *et al.*, 2019; Fujii *et al.*, 2022). Genes associated with the reductive pentose phosphate cycle (Calvin cycle) were also incomplete.

Complexes I–IV were largely absent, indicating that oxidative phosphorylation was generally not functional. However, some members of “*Ca.* Saccharimonadia”, “*Ca.* Doudnaibacteriota” (synonym “*Ca.* Doudnabacteria”), and other *Minisyncoccota* lineages retained partial complexes III–IV. “*Ca.* Saccharimonadia” has been reported to encode complex III, which may be involved in oxygen scavenging (Kantor *et al.*, 2013; Starr *et al.*, 2018; Lemos *et al.*, 2019; Kagemasa *et al.*, 2025). Lemos *et al.* (2019) further suggested that “*Ca.* Saccharimonadia”, although primarily reliant on fermentative metabolism, occasionally performs aerobic respiration. In this process, NAD⁺ is regenerated via a membrane-bound NADH dehydrogenase, which transfers electrons to ubiquinone. Ubiquinone then delivers electrons to cytochrome *o* ubiquinol oxidase, where oxygen is reduced to water, concurrently generating a proton motive force across the cytoplasmic membrane that drives ATP synthesis by ATP synthase (Lemos *et al.*, 2019). In contrast, complex V (ATPase), either the F or V/A type, was frequently retained, suggesting that ATP synthesis via proton motive force—or, conversely, ion transport coupled to ATP hydrolysis—remains possible.

## Fermentation, short-chain fatty acid metabolism, and other metabolic pathways

Genes encoding enzymes related to acetate production (acetate kinase and acetate-CoA ligase) were occasionally present, indicating that partial fermentative pathways operate in some lineages. In contrast, genes required for butyrate and propionate fermentation (butyrate kinase, propionate kinase, and propionate CoA-transferase) were largely absent, suggesting that typical butyrate- and propionate-producing fermentation is unlikely (Castelle *et al.*, 2018; Lemos *et al.*, 2020; Ji *et al.*, 2022; Maatouk *et al.*, 2023). However, “*Ca.* Parcunitrobacter nitroensis” has been reported to harbor genes associated with propionate fermentation (Castelle *et al.*, 2017). Genes for lactate fermentation (l- and d-lactate dehydrogenase) were frequently detected, indicating that lactate fermentation constitutes one of the major energy metabolism pathways (Danczak *et al.*, 2017; Hosokawa *et al.*, 2021; Chiriac *et al.*, 2022; Kuroda *et al.*, 2023b; Kagemasa *et al.*, 2025). Additional enzymes, such as formate C-acetyltransferase, were also partially retained, potentially enabling glycolysis-linked fermentation.

Genes required for the synthesis of amino acids, phospholipids, and nucleic acids were generally incomplete, suggesting that these compounds cannot be synthesized *de novo*. In contrast, genes for peptidoglycan biosynthesis were frequently observed, and in some bacteria, the complete pathway was identified (Castelle *et al.*, 2018). Therefore, the autonomous biosynthetic capacity appears to be extremely limited, implying strong dependence on host- or symbiont-derived metabolites.

Although metagenomic studies on subsurface and sedimentary communities indicated that *Minisyncoccota* contribute to nitrogen and sulfur cycling (Anantharaman *et al.*, 2016b; Danczak *et al.*, 2017; He *et al.*, 2021), detailed findings are not shown in this review. However, complete pathways are absent, with only one or two genes typically being present. Therefore, they are unlikely to perform multi-step redox transformations independently, instead occupying auxiliary roles within division-of-labor metabolic networks (Anantharaman *et al.*, 2016b; He *et al.*, 2021).

## Additional features common to *Minisyncoccota*

Genes encoding ComEC, a protein involved in DNA uptake, were found in most MAGs, while ComEA was present in some. These findings suggest the potential for natural transformation and the acquisition of exogenous DNA. Nearly all members harbored the core gene set for type IV pili (T4P), which appears to be a hallmark of *Minisyncoccota* (Castelle *et al.*, 2018; Lemos *et al.*, 2020; Ji *et al.*, 2022; Maatouk *et al.*, 2023). T4P are known to mediate host recognition, adhesion, and DNA uptake, thereby potentially compensating for their limited metabolic repertoire (Méheust *et al.*, 2019). Transmission and scanning electron microscopic observations have consistently revealed the presence of pili-like structures, further supporting T4P being a shared feature across this phylum (Kuroda *et al.*, 2022a, 2022b; Xie *et al.*, 2022; Yakimov *et al.*, 2022). Furthermore, isolate TM7i was shown to use pili for host attachment via twitching-like motility (Xie *et al.*, 2022). In addition, in *N. lyticus* TM7x, it has been experimentally demonstrated that two functionally distinct type IV pili are produced, with one mediating initial attachment to the host‍ ‍bacterium and the other driving twitching motility (Grossman *et al.*, 2025). Therefore, T4P-based episymbiotic relationships appear to be common within the phylum *Minisyncoccota*. In contrast, flagellar-related genes were not detected, and consistent with previous findings, they appear to be largely absent in the phylum *Minisyncoccota* (Castelle *et al.*, 2018). Collectively, these findings indicate that T4P play a crucial role in motility and host interactions within this lineage.

Host-associated lifestyles are common in the phylum *Minisyncoccota*. In contrast, a distinct genomic feature has been identified in the “*Ca.* Gracilibacteria”/JAEDAM01 lineage. While UGA functions as a stop codon in the standard genetic code, UGA-to-glycine recoding has been reported in this lineage (Rinke *et al.*, 2013). Single-cell genomic anal­yses revealed that in the lineage formerly known as SR1, many genes contained UGA codons at positions conserved for glycine residues, and also that a specialized tRNA^Gly^_UCA_ was aminoacylated with glycine by glycyl-tRNA synthetase (Campbell *et al.*, 2013). Large-scale metagenomic anal­yses have further demonstrated that UGA-to-glycine recoding is restricted to the “*Ca.* Gracilibacteria”/JAEDAM01 lineage, with its origin being traceable to their last common ancestor (Ivanova *et al.*, 2014).

## Conclusions

We herein provided an overview of current knowledge of the phylum *Minisyncoccota*, with a focus on historical transitions in its phylogenetic classification. We also summarized findings on PCR primer design, FISH probe development, metabolic features, phylogenetic diversity, and parasitic relationships. Members of *Minisyncoccota* exhibit extremely limited metabolic capacities, with the majority of central metabolic and cofactor biosynthetic pathways being absent or incomplete. Consistent with MAG-based metabolic inferences from both environmental samples and cultivated strains, they are generally unable to survive independently and are considered to adopt epibiotic lifestyles that are dependent on other microorganisms. T4P, frequently observed across this lineage, appear to mediate host recognition and attachment and may also contribute to DNA uptake, potentially facilitating survival through host interactions despite restricted metabolic repertoires. These findings suggest that *Minisyncoccota* are not merely metabolically constrained bacteria, but instead represent organisms that fulfill dependent or interdependent roles within microbial communities. Overall, our understanding of microbial diversity and evolution has been expanded by the phylum *Minisyncoccota*, and its existence provides insights into the “hidden” division of labor within microbial ecosystems. Future efforts, particularly the isolation of additional uncultivated strains and experimental validation, will be essential to further elucidate the functional roles and symbiotic mechanisms of *Minisyncoccota*.

## Citation

Fujii, N., Nakajima, M., Narihiro, T., Kuroda, K., and Kindaichi, T. (2026) Current Understanding of Taxonomy and Ecology of the Phylum *Minisyncoccota*. *Microbes Environ ***41**: ME25084.

https://doi.org/10.1264/jsme2.ME25084

## Supplementary Material

Supplementary Material 1

Supplementary Material 2

## Figures and Tables

**Table 1.** Phylogenetic Correspondences

Former name			Current names under ICNP			Current names under SeqCode			Current names in databases		
Shorter	Longer		Name	Status		Name	Status		GTDB	SILVA 138.2	MiDAS 5.3
TM7	*Saccharibacteria*		“*Ca.* Saccharimonadia”	pro-validly		N/A	N/A		*Saccharimonadia*	*Saccharimonadia*	*Saccharimonadia*
OD1	*Parcubacteria*/*Paceibacteria*		*Minisyncoccia*	validly		*Minisyncoccia*	Valid (ICNP)		*Minisyncoccia*	*Parcubacteria*	*Parcubacteria*
			“*Ca.* Patescibacteriia”	not pro-validly		*Patescibacteriia*	Valid (SeqCode)		*Patescibacteriia*	ABY1	ABY1
OP11	*Microgenomates*		“*Ca.* Microgenomatia”	not pro-validly		“Microgenomatia”	Automated discovery		*Microgenomatia*	*Microgenomatia*	*Microgenomatia*
BD1-5/GN02	*Gracilibacteria*		“*Ca.* Gracilibacteria”	not pro-validly		“Gracilibacteria”	Draft		JAEDAM01	*Gracilibacteria*	*Gracilibacteria*
SR1 (ACD80)	*Absconditabacteria*		“*Ca.* Gracilibacteria”	not pro-validly		“Gracilibacteria”	Draft		JAEDAM01	*Gracilibacteria*	*Gracilibacteria*
PER	*Peregrinibacteria*		“*Ca.* Gracilibacteria”	not pro-validly		“Gracilibacteria”	Draft		*Gracilibacteria*	*Gracilibacteria*	*Gracilibacteria*
WS6	*Dojkabacteria*		“*Ca.* Dojkaibacteriota”	pro-validly		“Dojkabacteria”	Automated discovery		*Dojkabacteria*	*Dojkabacteria*	*Dojkabacteria*
WWE3	*Katanobacteria*		“*Ca.* Katanibacteriota”	pro-validly		N/A	N/A		WWE3	WWE3	WWE3
ACD58	*Berkelbacteria*		“*Ca.* Berkeliibacteriota”	pro-validly		N/A	N/A		UBA1384	*Berkelbacteria*	*Berkelbacteria*
SM2F11	*Doudnabacteria*		“*Ca.* Doudnaibacteriota”	pro-validly		“Doudnabacteria”	Automated discovery		*Doudnabacteria*	*Parcubacteria*	*Parcubacteria*
N/A	*Andersenbacteria*		“*Ca.* Anderseniibacteriota”	pro-validly		“Andersenbacteria”	Automated discovery		*Andersenbacteria*	N/A	N/A
N/A	Kazan		N/A	not available		N/A	N/A		Kazan-3B-28	Kazania	Kazania

N/A: Not Available. Validly: the nomenclatural status is validly published under the ICNP. Pro-validly: the nomenclatural pro-status is pro-validly published under the ICNP. Not pro-validly: the nomenclatural pro-status is not pro-validly published. Valid (ICSP): this name has been validly published under the rules of the ICNP and has priority in the scientific record. Valid (SeqCode): this name has been validly published under the rules of SeqCode and has priority in the scientific record. Draft/Automated discovery: this name was automatically created by the system and has not undergone expert review.

**Table 2.** Morphological characteristics and cell dimensions of members of the phylum *Minisyncoccota*

Species	Shape	Length (nm)^*1^	Width (nm)^*1^	Reference
“*Ca.* Sonnebornia yantaiensis”	rod	1,600–1,900	500–600	Gong *et al.*, 2014
“*Ca.* Nanosynbacter lyticus”	coccoid	200–500	200–500	He *et al.*, 2015; Bor *et al.*, 2016, 2018; Utter *et al.*, 2020
“*Ca.* Nanosynbacter” sp.	coccoid/ovoid	100–300	100–300	Ibrahim *et al.*, 2021
“*Ca.* Mycolatisynbacter gordoniilyticus”	ovoid	424–478	224–264	Batinovic *et al.*, 2021
“*Ca.* Vampirococcus lugosii”	flat	200–240	500–600	Moreira *et al.*, 2021
“*Ca.* Absconditicoccus praedator”	coccoid	419–572	419–572	Yakimov *et al.*, 2022
“*Ca.* Yanofskyibacterium parasiticum”	coccoid	330–590	290–430	Kuroda *et al.*, 2022b, 2024
*Minisyncoccus archaeiphilus*	coccoid	630–830	400–520	Kuroda *et al.*, 2022a, 2024; Nakajima *et al.*, 2025
“*Ca.* Microsyncoccus archaeolyticus”	coccoid	330–510	240–320	Kuroda *et al.*, 2022a, 2024
“*Ca.* Microsaccharimonas” sp.	coccoid	219–567	136–apx.300^*2^	Xie *et al.*, 2022
“*Ca.* Nealsonbacteria” sp.	coccoid	200–800	200–800	Chen *et al.*, 2023

*1 When “diameter” was reported in the original publication, the same value was used for both length and width for consistency. *2 Estimated from figures in the original publication
