# Supplementary material for: Current Understanding of Taxonomy and Ecology of the Phylum Minisyncoccota
Source: Microbes Environ. 2026 May 12;41(2):ME25084. doi: 10.1264/jsme2.ME25084 (PMC13293708; doi:10.1264/jsme2.ME25084)
Supplement: Supplementary file 1 — Supplementary Material 1 [file 41_25084_s1.pdf]

# Current Understanding of Taxonomy and Ecology of the Phylum *Minisyncoccota*

## -Supplemental Information and Supplemental Tables-

**Naoki Fujii[1], Meri Nakajima[2], Takashi Narihiro[2,3], Kyohei Kuroda[2,3*], Tomonori Kindaichi[1,4*]**

[1]Department of Civil and Environmental Engineering, Graduate School of Advanced Science and Engineering, Hiroshima University, 1-4-1, Kagamiyama, Higashihiroshima, Hiroshima 739-8527, Japan;
[2]Biomanufacturing Process Research Center, National Institute of Advanced Industrial Science and Technology (AIST), 2-17-2-1 Tsukisamu-Higashi, Toyohira-ku, Sapporo, Hokkaido 062-8517, Japan;
[3]Integrated Research Center for Circular Technology, National Institute of Advanced Industrial Science and Technology (AIST), 2-17-2-1 Tsukisamu-Higashi, Toyohira-ku, Sapporo, Hokkaido, 062-8517 Japan;
[4]Dairy Ecosystem Research & Development Center, Hiroshima University, 2-2965, Kagamiyama, Higashi-Hiroshima, Hiroshima, 739-0046, Japan

***Correspondence:**
Tomonori Kindaichi; Tel: +81-82-424-5718; E-mail: tomokin@hiroshima-u.ac.jp
Kyohei Kuroda; Tel: +81-50-3522-8902; E-mail: k.kuroda@aist.go.jp

## Materials and Methods

### Retrieval and Filtering of MAGs

A total of 10,847 MAGs (metagenome-assembled genomes) registered in the GTDB Release 226 (Parks *et al.*, 2025) were targeted. Among them, 10,798 MAGs were successfully downloaded from GenBank. Filtering was performed according to the criteria for high-quality MAGs defined by MIMAG (completeness ≥ 90% and contamination ≤ 5%), resulting in the retention of 2,846 MAGs (Bowers *et al.*, 2017). Additional filtering was conducted, resulting in a final selection of 420 genomes.

### Additional Filtering of MAGs

To obtain a more reliable set of genomes, additional filtering was conducted under the following criteria:

1. Completeness and Contamination

   MAGs with relatively high completeness or contamination values were reviewed in subsets of five.

2. Taxonomic Verification

   ･Only genomes designated as gtdb_species_representative were retained.

   ･MAGs with inconsistent taxonomy between NCBI and GTDB (*e.g.*, annotated as Paceibacteria in NCBI but as Saccharimonadia in GTDB) were excluded.

3. Presence of 16S rRNA Genes

   Genomes containing 16S rRNA genes were retained for subsequent comparison with the SILVA and MIDAS databases.

4. Coverage of Taxonomic Diversity

   MAGs were selected to maximize the inclusion of diverse lineages in GTDB. Even if genus-level distinctions were not available, representation at the family level was ensured whenever possible.

5. Quality Evaluation Metrics

   MAGs were further evaluated by genome size, completeness/contamination ratio, presence of 23S/5S/tRNA genes, and number of contigs. Genomes not meeting these standards were excluded, but unrepresented lineages were included for reference.

### Genome annotation and additional analysis

Genomes were annotated using a combination of Prokka v1.13.0 (Seemann, 2014), DRAM v1.5 (Shaffer *et al.*, 2020), and the MacsyFinder TFFScan or TXXScan model (Abby *et al.*, 2016). The annotation results obtained from DRAM and MacsyFinder were subsequently visualized as multi-symbol annotation map using R (R Core Team, 2023). To verify the phylogenetic placement of 16S rRNA genes, we followed an analysis workflow adapted from (Brown *et al.*, 2015). The covariance models from Rfam (16S: RF00177; 23S: RF02541; 5S: RF00001, if required) were employed, and Infernal (cmsearch) was used to identify 16S rRNA gene regions from MAG sequences (Nawrocki *et al.*, 2015). The detected coordinates were subsequently used to extract the corresponding regions from FASTA files. In cases with duplicate hits or multiple copies,

representative sequences were selected by considering annotation consistency (*e.g.*, rRNA/tRNA predictions) and sequence completeness. The resulting 16S sequences longer than 800 bp were structurally aligned using SSU-ALIGN v0.1.1 with the bacterial covariance model (Nawrocki, 2009), and the alignments were converted into FASTA format. Following the procedure of Brown *et al.* (2015), insertions exceeding 10 bp (determined by comparison with *Escherichia coli* K-12) were removed to ensure alignment reliability. Columns consisting only of gaps or poorly aligned regions were also discarded to refine the dataset. Where necessary, insertions were manually inspected and corrected using visualization software. The recovered 16S rRNA genes were taxonomically classified using QIIME2 v2024.10 (Bolyen *et al.*, 2019) with the MiDAS v5.3 (Nierychlo *et al.*, 2020) and SILVA v138.2 (Quast *et al.*, 2013) reference databases. The phylogenetic trees were constructed using GTDB-Tk v2.2.6 (Chaumeil *et al.*, 2019) and IQ-TREE v2.2.2.3 (Minh *et al.*, 2020), following the protocol described in Nakajima *et al.* 2025.

**References**

Abby, S.S., Cury, J., Guglielmini, J., Néron, B., Touchon, M. and Rocha, E.P.C. (2016) Identification of protein secretion systems in bacterial genomes. *Sci Rep* **6**: 23080.

Albertsen, M., Hugenholtz, P., Skarshewski, A., Nielsen, K.L., Tyson, G.W., and Nielsen, P.H. (2013) Genome sequences of rare, uncultured bacteria obtained by differential coverage binning of multiple metagenomes. *Nat Biotechnol* **31**: 533−538.

Batinovic, S., Rose, J.J.A., Ratcliffe, J., Seviour, R.J. and Petrovski, S. (2021) Cocultivation of an ultrasmall environmental parasitic bacterium with lytic ability against bacteria associated with wastewater foams. *Nat Microbiol* **6**: 703−711.

Bolyen, E., Rideout, J.R., Dillon, M.R., Bokulich, N.A., Abnet, C.C., Al-Ghalith, G.A. *et al.*, (2019) Reproducible, interactive, scalable and extensible microbiome data science using QIIME 2. *Nat Biotechol*, **37**, 852–857.

Bor, B., Poweleit, N., Bois, J.S., Cen, L., Bedree, J.K., Hong Zhou, Z., *et al.*, (2016) Phenotypic and Physiological Characterization of the Epibiotic Interaction Between TM7x and Its Basibiont Actinomyces. *Microb Ecol* **71**: 243−255.

Bor, B., McLean, J.S., Foster, K.R., Cen, L., To, T.T., Serrato-Guillen, A., *et al.*, (2018) Rapid evolution of decreased host susceptibility drives a stable relationship between ultrasmall parasite TM7x and its bacterial host. *Proc Natl Acad Sci USA*, **115**, 12277−12282.

Bor, B., Collins, A.J., Murugkar, P.P., Balasubramanian, S., To, T.T., Hendrickson, E.L., *et al.*, (2020) Insights Obtained by Culturing Saccharibacteria With Their Bacterial Hosts. *J Dent Res* **99**: 685−694.

Borrel, G., Lehours, A.C., Bardot, C., Baily, X. and Fonty, G. (2010) Members of candidate divisions OP11, OD1 and SR1 are widespread along the water column of the meromictic Lake Pavin (France) *Arch Microbiol* **192**: 559–567.

Bowers, R.M., Krypides, N.C., Stepanauskas, R., Harmon-Smith, M., Doud, D., Reddy, T.B.K., *et al*., (2017) Minimum information about a single amplified genome (MISAG) and a metagenome-assembled genome (MIMAG) of bacteria and archaea. *Nat Biotechol* **35**: 725–731.

Brinig, M.M., Lepp, P.W., Ouverney, C.C., Armitage, G.C. and Relmn, D.A. (2003) Prevalence of Bacteria of Division TM7 in Human Subgingival Plaque and Their Association with Disease. *Appl Environ Microbiol* **69**: 1687−1694.

Brown, C.T., Hug, L.A., Thomas, B.C., Sharon, I., Castelle, C.J., Singh, A., *et al*., (2015) Unusual biology across a group comprising more than 15% of domain Bacteria. *Nature* **523**: 208−211.

Camanocha, A. and Dewhirst, F.E. (2014) Host-associated bacterial taxa from Chlorobi, Chloroflexi, GN02, Synergistetes, SR1, TM7, and WPS-2 Phyla/candidate divisions *J Oral Microbiol* **6**: 25468.

Chaumeil, P.A., Mussig, A.J., Hugenholtz, P. and Parks, D.H. (2019) GTDB-Tk: a toolkit to classify genomes with the Genome Taxonomy Database. *Bioinformatics* **36**: 1925–1927.

Chen, X., Molenda, O., Brown, C.T., Toth, C.R.A., Guo, S., Luo, F., *et al*., (2023) "Candidatus Nealsonbacteria" Are Likely Biomass Recycling Ectosymbionts of Methanogenic Archaea in a Stable Benzene-Degrading Enrichment Culture. *Appl Environ Microbiol* **89**: 00025-23.

Chiriac, M.C., Bulzu, P.A., Andrei, A.S., Okazaki, Y., Nakano, S., Haber, M., *et al*., (2022) Ecogenomics sheds light on diverse lifestyle strategies in freshwater CPR. *Microbiome* **10**: 84.

Chipashvili, O., Utter, D.R., Bedree, J.K., Ma, Y.S., Schulte, F., Mascarin, G., *et al*., (2021) Episymbiotic Saccharibacteria suppresses gingival inflammation and bone loss in mice through host bacterial modulation. *Cell Host Microbe* **29**: 1649−1662.

Cross, K.L., Campbell, J.H., Balachandran, M., Campbell, A.G., Cooper, C.J., Griffen, A., *et al*., (2019) Targeted isolation and cultivation of uncultivated bacteria by reverse genomics. *Nat Microbiol* **37**: 1314−1321.

Dinis, J.M., Barton, D.E., Gharidi, J., Surendar, D., Reddy, K., Velasquez, F., *et al*., (2011) In Search of an Uncultured Human-Associated TM7 Bacterium in the Environment. *PLoS One* **6**: e21280.

Ferrari, B., Winsley, T., Ji, M. and Neilan, B. (2014) Insights into the distribution and abundance of the ubiquitous Candidatus Saccharibacteria phylum following tag pyrosequencing. *Sci Rep* **4**:3957.

Fujii, N., Kuroda, K., Narihiro, T., Aoi, Y., Ozaki, N., Ohashi, A., *et al*., (2024) Unique episymbiotic relationship between Candidatus Patescibacteria and Zoogloea in activated sludge flocs at a municipal wastewater treatment plant. *Environ Microbiol Rep* **16(5)**: e70007.

Gong, J., Qing, Y., Guo, X.H. and Warren, A. (2014) "Candidatus Sonnebornia yantaiensis", a member of candidate division OD1, as intracellular bacteria of the ciliated protist Paramecium bursaria (Ciliophora, Oligohymenophorea). *Syst Appl Microbiol* **37**: 35−41.

Guermazi, S., Daegelen, P., Dauga, C., Rivière, D., Bouchez, T., Godon, J.J., *et al*., (2008) Discovery and characterization of a new bacterial candidate division by an anaerobic sludge digester metagenomic approach. *Environ Microbiol* **10**: 2111−2123.

Hanke, A., Hamann, E., Sharma, R., Geelhoed, J.S., Hargesheimer, T., Kraft, B., *et al*., (2014) Recoding of the stop codon UGA to glycine by a BD1-5/SN-2 bacterium and niche partitioning between Alpha- and Gammaproteobacteria in a tidal sediment microbial community naturally selected in a laboratory chemostat. *Front Microbiol* **5**: 231.

Harris, J.K., Kelley, S.T. and Pace, N.R. (2004) New Perspective on Uncultured Bacterial Phylogenetic Division OP11. *Appl Environ Microbiol* **70**: 845–849.

He, C., Keren, R., Whittaker, M.L., Farag, I.F., Doudna, J.A., Cate, J.H.D., *et al*., (2021) Genome-resolved metagenomics reveals site-specific diversity of episymbiotic CPR bacteria and DPANN archaea in groundwater ecosystems. *Nat commun* **6**: 354–365.

Hu, H., Kristensen, J.M., Herbold, C.W., Pjevac, P., Kitzinger, K., Hausmann, B., *et al*., (2024) Global abundance patterns, diversity, and ecology of Patescibacteria in wastewater treatment plants. *Microbiome* **12**: 55.

Hugenholtz, P., Tyson, G.W., Webb, R.I., Wagner, A.M. and Blackall, L.L. (2001) Investigation of Candidate Division TM7, a Recently Recognized Major Lineage of the Domain Bacteria with No Known Pure-Culture Representatives. *Appl Environ Microbiol* **67**: 411–419.

Ibrahim, A., Maatouk, M., Rajaonison, A., Zgheib, R., Haddad, G., Khalil, J.B., *et al*., (2021) Adapted Protocol for Saccharibacteria Cocultivation: Two New Members Join the Club of Candidate Phyla Radiation. *Microbiol Spectr* **9**: e01069-21.

Kuroda, K., Yamamoto, K., Nakai, R., Hirakata, Y., Kubota, K., Nobu M.K., *et al*., (2022A) Symbiosis between Candidatus Patescibacteria and Archaea Discovered in Wastewater-Treating Bioreactors. *mBio* **13**: 01711-22.

Kuroda, K., Kubota, K., Kagemasa, S., Nakai, R., Hirakata, Y., Yamamoto, L., *et al*., (2022B) Novel Cross-domain Symbiosis between Candidatus Patescibacteria and Hydrogenotrophic Methanogenic Archaea Methanospirillum Discovered in a Methanogenic Ecosystem. *Microbes Environ* **37**:ME22063.

Kuroda, K., Tomita, S., Kurashita, H., Hatamoto, M., Yamaguchi, T., Hori, T., Aoyagi, T., Sato, Y., Inaba, T., Habe, H., Tamaki, H., Hagihara, Y., Tamura, T., Narihiro, T. (2023B) Metabolic implications for predatory and parasitic bacterial lineages in activated sludge wastewater treatment systems. *Water Res X* **20**:100196.

Man D.K.W. Hermans, S.M., Taubert, M., Garcia, S.L., Hengoju, S., Küsel, K., *et al*., (2024) Enrichment of different taxa of the enigmatic candidate phyla radiation bacteria using a novel picolitre droplet technique. *ISME commun* **4**: ycae080.

McLean, J.S., Bor, B., Kerns, K.A., Liu, Q., To, T.T., Solden, L., *et al*., (2020) Acquisition and Adaptation of Ultra-small Parasitic Reduced Genome Bacteria to Mammalian Hosts. *Cell Rep* **32**:107939.

Minh, B.Q., Schmidt, H.A., Chernomor, O., Schrempf, D., Woodhams, M.D., Von Haeseler, A. *et al*., (2020) IQ-TREE 2: new models and efficient methods for phylogenetic inference in the genomic era. *Mol Biol Evol* **37**: 1530–1534.

Moreira, D., Zivanovic, Y., López-Archilla, A.I., Iniesto, M. and López-García, P. (2021) Reductive evolution and unique predatory mode in the CPR bacterium *Vampirococcus lugosii*. *Nat Commun* **12**: 2454.

Murugkar, P.P., Collins, A.J., Chen, T. and Dewhirst F.E. (2020) Isolation and cultivation of candidate phyla radiation *Saccharibacteria* (TM7) bacteria in coculture with bacterial hosts. *J Oral Microbiol* **12**:1814666.

Nakajima, M., Nakai, R., Hirakata, Y., Kubota, K., Satoh, H., Nobu, M.K., *et al.,* (2025) *Minisyncoccus archaeiphilus* gen. nov., sp. nov., a mesophilic, obligate parasitic bacterium and proposal of *Minisyncoccaceae* fam. nov., *Minisyncoccales* ord. nov., Minisyncoccia class. nov. and *Minisyncoccota* phyl. nov. formerly referred to as *Candidatus* Patescibacteria or candidate phyla radiation. *Int J Syst Evol Microbiol* **75**: 6668.

Nawrocki, E.P. (2009) Structural RNA homology search and alignment using covariance models. Ph.D. Thesis, Washington University in St. Louis.

Nawrocki, E.P., Burge, S.W., Bateman, A., Daub, J., Eberhardt, R.Y., Eddy, S.R., *et al*., (2015) Rfam 12.0: updates to the RNA families database. *Nucleic Acids Res* **43**: D130–D137.

Nie, J., Utter, D.R., Kerns, K.A., Lamont, E.I., Hendrickson, E.L., Liu, J., *et al*., (2022) Strain-Level Variation and Diverse Host Bacterial Responses in Episymbiotic Saccharibacteria. *mSystems* **7**: e01488-21.

Nierychlo, M., Andersen, K.S., Xu, Y., Green, N., Jian, C.J., Albertsen, M., *et al*., (2020) MiDAS 3: An ecosystem-specific reference database, taxonomy and knowledge platform for activated sludge and anaerobic digesters reveals species-level microbiome composition of activated sludge. *Water Res* **182**: 115955.

Nittami, T., Speirs L.B.M., Fukuda, J., Watanabe, M. and Seviour R.J. (2014) Fluorescence in situ hybridization probes targeting members of the phylum Candidatus Saccharibacteria falsely target Eikelboom type 1851 filaments and other Chloroflexi members. *Environ Microbiol Rep* **6**: 611−617.

Parks, D.H., Chaumeil, P.A., Mussig, A.J., Rinke, C., Chuvochina, M., Hugenholtz, P. (2025) GTDB release 10: a complete and systematic taxonomy for 715 230 bacterial and 17 245 archaeal genomes. *Nucleic Acids Res* gkaf1040.

Quast, C., Pruesse, E., Yilmaz, P., Gerken, J., Schweer, T., Yarza, P. *et al*., (2013) The SILVA ribosomal RNA gene database project: improved data processing and web-based tools. *Nucleic Acids Research,* **41**, D590–D596.

R Core Team (2023) R: A language and environment for statistical computing. R Foundation for Statistical Computing, Vienna, Austria. https://www.R-project.org/

Seemann, T. (2014) Prokka: rapid prokaryotic genome annotation. *Bioinformatics*, **30**, 2068–2069.

Shaffer, M., Borton, M.A., McGivern, B.B., Zayed, A.A. La Rosa, S.L., Solden, L.M., *et al*., (2020) DRAM for distilling microbial metabolism to automate the curation of microbiome function. *Nucleic Acids Res*, **48**, 8883–8900.

Singleton, C.M., Petriglier, F., Kristensen, J.M. Kirkegaard, R.H. Michaelsen, T.Y., *et al*., (2021) Connecting structure to function with the recovery of over 1000 high-quality metagenome-assembled genomes from activated sludge using long-read sequencing. *Nat Commun* **12**: 2009.

Utter, D.R., He, X., Cavanauph, C.M., McLean, J.S. and Bor, B. (2020) The saccharibacterium TM7x elicits differential responses across its host range. *ISME J* **14**: 3054−3067.

Xie, B., Wang, J., Nie, Y., Tian, J., Wang, Z., Chen, D. *et al*., (2022) Type IV pili trigger episymbiotic association of Saccharibacteria with its bacterial host. *Proc Natl Acad Sci USA,* **119**, e2215990119.

Yakimov, M.M., Merkel, A.Y., Gaisin, V.A., Pilhofer, M., Messina, E., Hallsworth, J.E., *et al*., (2021) Cultivation of a vampire: '*Candidatus* Absconditicoccus praedator'. *Environ Microbiol* **24**: 30–49.

Yang Y.W., Chen, M.K., Yang B.Y., Huang, X.J., Zhang, X.R., He, L.Q. *et al*., (2015) Use of 16S rRNA Gene-Targeted Group-Specific Primers for Real-Time PCR Analysis of Predominant Bacteria in Mouse Feces. *Appl Environ Microbiol* **81**: 6749−6756.

## Supplemental Tables

**Table S1**. List of qPCR Primers

| Primer name | Lineage | Target | Outgroup | SSU type | Orientation | Sequence (5'→3') | Reference |
|---|---|---|---|---|---|---|---|
| TM7314F | "*Ca.* Saccharimonadia" | 568 | 303 | 16S | Forward | GAG AGG ATG ATC AGC CAG | Hugenholtz *et al*., 2001 |
| TM7580F | "*Ca.* Saccharimonadia" | 992 | 28 | 16S | Forward | AYT GGG CGT AAA GAG TTG C | Hugenholtz *et al*., 2001 |
| TM7-910F | "*Ca.* Saccharimonadia" | 997 | 12 | 16S | Forward | CAT AAA GGA ATT GAC GGG GAC | Brinig *et al*., 2003 |
| TM7-1177R | "*Ca.* Saccharimonadia" | 860 | 138 | 16S | Reverse | GAC CTG ACA TCA TCC CCT CCT TCC | Brinig *et al*., 2003 |
| TM7-1093F | "*Ca.* Saccharimonadia" | 827 | 27 | 16S | Forward | AGT CCA TCA ACG AGC GCA ACC | Brinig *et al*., 2003 |
| I025-135F | "*Ca.* Saccharimonadia" | 4 | 0 | 16S | Forward | CCC TGC AGT GAG GGA TAA GA | Brinig *et al*., 2003 |
| I025-590R | "*Ca.* Saccharimonadia" | 5 | 0 | 16S | Reverse | GTT TTC ATC GCT CGC TAA CTT G | Brinig *et al*., 2003 |
| 11_1_502F | "*Ca.* Miccrogenomatia" | 122 | 2 | 16S | Forward | GCC GCT AAC TGC GTG CCA | Harris *et al*., 2004 |
| 11_2_1286R | "*Ca.* Miccrogenomatia" | 62 | 1 | 16S | Reverse | CCC ACT GAG AAG CCG TTT | Harris *et al*., 2004 |
| 11_3_1368R | "*Ca.* Miccrogenomatia"<br>*Minisyncoccia* | 269<br>6 | 19 | 16S | Reverse | GCG AGA ACG TAT TCA CCG | Harris *et al*., 2004 |
| 11_5_1380R | "*Ca.* Gracilibacteria" | 137 | 9 | 16S | Reverse | TGA GTG CAA GGA ACA GGG | Harris *et al*., 2004 |
| OP11-1090R | "*Ca.* Miccrogenomatia" | 168 | 1 | 16S | Reverse | TCG TTG TCC CAC TTA A | Harris *et al*., 2004 |
| WWE3-1322R | WWE3 ("*Ca.* Katanibacteriota") | 8 | 1 | 16S | Reverse | CTT TGC TGA CGT GAC GGG | Guermazi *et al*., 2008 |
| WWE3-289F | WWE3 ("*Ca.* Katanibacteriota") | 24 | 3862 | 16S | Forward | GGG CAC TGA GAC ACG GG | Guermazi *et al*., 2008 |
| WWE3-948R | WWE3 ("*Ca.* Katanibacteriota") | 13 | 1 | 16S | Reverse | TGG ATA CCG GTC GTT CC | Guermazi *et al*., 2008 |
| WWE3-149F | WWE3 ("*Ca.* Katanibacteriota") | 4 | 0 | 16S | Forward | GGC GGG GTA ATT CCT TAT | Guermazi *et al*., 2008 |
| WWE3-1202R | WWE3 ("*Ca.* Katanibacteriota") | 5 | 0 | 16S | Reverse | CTG AGA GGT CGT TTA GCG | Guermazi *et al*., 2008 |
| WWE3-21DF | WWE3 ("*Ca.* Katanibacteriota") | 9 | 6 | 16S | Forward | GGN TCA GGG TGA ATG CTA | Guermazi *et al*., 2008 |
| WWE3-1282DR | WWE3 ("*Ca.* Katanibacteriota") | 40* | 1513* | 16S | Reverse | CRT ATT CAC SGN NGT ATA GCT G | Guermazi *et al*., 2008 |
| WWE3-ExtF | WWE3 ("*Ca.* Katanibacteriota") | 0 | 0 | 16S | Forward | GCA CTT TGA AAA GGT ATC CT | Guermazi *et al*., 2008 |
| WWE3-ExtR | WWE3 ("*Ca.* Katanibacteriota") | 0 | 0 | 16S | Reverse | CCT ACT CAA CTG TTT GTG AG | Guermazi *et al*., 2008 |
| WWE3-21F | WWE3 ("*Ca.* Katanibacteriota") | 5 | 0 | 16S | Forward | GGT TCA GGG TGA ATG CTA | Guermazi *et al*., 2008 |
| 907f-SR1 | "*Ca.* Gracilibacteria" | 180 | 1 | 16S | Forward | AAA CTC AAA GGG ATA GGC GG | Borrel *et al*., 2010 |
| TM7a-997F | "*Ca.* Saccharimonadia" | 15 | 0 | 16S | Forward | TCC CGA GAA GAT TTA CG | Dinis *et al*., 2011 |
| HUM-TM7a-1112R | "*Ca.* Saccharimonadia" | 14 | 0 | 16S | Reverse | ACA ACT AGA CAC AAG GG | Dinis *et al*., 2011 |
| ENV-TM7a-1112R | "*Ca.* Saccharimonadia" | 27 | 0 | 16S | Reverse | TCA ACT ATT CAC AAG GG | Dinis *et al*., 2011 |
| TM7-211F | "*Ca.* Saccharimonadia" | 801 | 79 | 16S | Forward | GAG CGG CGG ACG GCT GAG | Ferrari *et al*., 2014 |
| TM7-686R | "*Ca.* Saccharimonadia" | 1074* | 53* | 16S | Reverse | CTA CGC AAC YCT TTA CRC CC | Ferrari *et al*., 2014 |
| TM7-590F* | "*Ca.* Saccharimonadia" | 1067* | 22* | 16S | Forward | GWA AAG AGT WGC GTA GGY GG | Ferrari *et al*., 2014 |
| TM7-965R* | "*Ca.* Saccharimonadia" | 921* | 14* | 16S | Reverse | WTR CTT AAC GCG TTA GCT T CGC T | Ferrari *et al*., 2014 |
| AF25 | "*Ca.* Gracilibacteria" | 33 | 657 | 16S | Forward | CTG GCT CAG GGT GAA C | Camanocha *et al*., 2014 |
| AF29 | "*Ca.* Gracilibacteria" | 164 | 47 | 16S | Forward | TGA ACG CTA GCG GTG C | Camanocha *et al*., 2014 |
| AF29-X1 | "*Ca.* Gracilibacteria" | 4 | 0 | 16S | Forward | GAT TAA YGC TAG CTG TGC | Camanocha *et al*., 2014 |
| AF26 | "*Ca.* Gracilibacteria" | 32 | 1 | 16S | Reverse | GTC AGT AGT CCT ACC ATG | Camanocha *et al*., 2014 |
| AF27 | "*Ca.* Gracilibacteria" | 18 | 1113 | 16S | Reverse | CCT TGT TAC GAC TTA AGC | Camanocha *et al*., 2014 |
| AF28 | "*Ca.* Gracilibacteria" | 26 | 65 | 16S | Reverse | CCT TGT TAC GAC TTC AGC | Camanocha *et al*., 2014 |
| AF33 | "*Ca.* Gracilibacteria"<br>"*Ca.* Saccharimonadia" | 39<br>314 | 3014 | 16S | Reverse | CCT TGT TAC GAC TTA ACC | Camanocha *et al*., 2014 |
| AF31 | "*Ca.* Gracilibacteria" | 13 | 1 | 16S | Forward | GAT GAA CGC TAG CGA AAT G | Camanocha *et al*., 2014 |
| AF31-X1 | "*Ca.* Gracilibacteria" | 47* | 3* | 16S | Forward | GAT GAA CGC TAG CGR AAY G | Camanocha *et al*., 2014 |
| AF32 | "*Ca.* Gracilibacteria" | 0 | 0 | 16S | Reverse | GTC ACT GGG GTT AAG CTT | Camanocha *et al*., 2014 |
| TM7-X1 | "*Ca.* Saccharimonadia" | 228 | 117635 | 16S | Forward | AGA GTT TGA TCC TGG CTC | Camanocha *et al*., 2014 |
| AH67 | "*Ca.* Gracilibacteria" | 29 | 66672 | 23S | Forward | AGT ACG AGA GGA CCG G | Camanocha *et al*., 2014 |
| AH68 | "*Ca.* Gracilibacteria" | 19 | 9723 | 23S | Forward | AAC CRC TGA AAG CAT CTA AG | Camanocha *et al*., 2014 |
| Sac1031F | "*Ca.* Saccharimonadia" | 40 | 1 | 16S | Forward | AAG AGA ACT GTG CCT TCG G | Yang *et al*., 2015 |
| Sac1218R | "*Ca.* Saccharimonadia" | 94 | 2 | 16S | Reverse | GCG TAA GGG AAA TAC TGA CC | Yang *et al*., 2015 |
| 400F | "*Ca.* Nanosynbacter lyticus" ("*Ca.* Saccharimonadia") | 176 | 0 | 16S | Forward | TAT GAG TGA AGA ATA TGA C | Bor *et al*., 2016 |
| 1110R | "*Ca.* Nanosynbacter lyticus" ("*Ca.* Saccharimonadia") | 31 | 0 | 16S | Reverse | CAG TCC AAG TAG AAA AAT AC | Bor *et al*., 2016 |
| 515F (V4) | modified V4 primer ("*Ca.* Saccharimonadia") | 1100 | 155 | 16S | Forward | GTG CCA GCM GCC GCG GTC A | Cross *et al*., 2019 |
| SR1-31F | "*Ca.* Gracilibacteria" | 13 | 1 | 16S | Forward | GAT GAA CGC TAG CGA AAT | Cross *et al*., 2019 |
| SR1-32R | "*Ca.* Gracilibacteria" | 6 | 4 | 16S | Reverse | CTT AAC CCC AGT CAC TGA TT | Cross *et al*., 2019 |

**Table S1**. Continued.

| Primer name | Lineage | Target | Outgroup | SSU type | Orientation | Sequence (5'→3') | Reference |
|---|---|---|---|---|---|---|---|
| AJ02 | "*Ca.* Saccharimonadia" | 260 | 46297 | 16S | Forward | ATC CTG GCT CAG GAT KAA | Murugkar *et al*., 2020 |
| AI85 | "*Ca.* Saccharimonadia" | 7 | 1 | 16S | Reverse | AAG GAG GTA ATC CAT CCG | Murugkar *et al*., 2020 |
| G3-F73 | "*Ca.* Saccharimonadia" | 4 | 0 | 16S | Forward | ACT TGG CTA TTA TGC GAG T | McLean *et al*., 2020 |
| G3-R1469 | "*Ca.* Saccharimonadia" | 56 | 3414 | 16S | Reverse | GGA TAC CTT GTT ACG AC | McLean *et al*., 2020 |
| G5-F47 | "*Ca.* Saccharimonadia" | 5 | 0 | 16S | Forward | GCT CTT CGG AGT ACA CGA GA | McLean *et al*., 2020 |
| G5-R1336 | "*Ca.* Saccharimonadia" | 2 | 0 | 16S | Reverse | AAA TAA ATC CGG ACG TCG GGT GCT CC | McLean *et al*., 2020 |
| G6-F180 | "*Ca.* Saccharimonadia" | 18 | 126 | 16S | Forward | GCA TCG AAA GGT GTG C | McLean *et al*., 2020 |
| G6-R1469 | "*Ca.* Saccharimonadia" | 315 | 3576 | 16S | Reverse | CCT TGT TAC GAC TTA AC | McLean *et al*., 2020 |
| SacchariF | "*Ca.* Saccharimonadia" | 2 | 11 | 23S | Forward | GGC TTA TAG CGC CCA ATA G | Ibrahim *et al*., 2021 |
| SacchariR | "*Ca.* Saccharimonadia" | 2 | 9 | 23S | Reverse | CGG ATA TAA ACC GAA CTG TC | Ibrahim *et al*., 2021 |
| Forward primer for M. amalyticus | "*Ca.* Mycolatisynbacter gordoniilyticus" ("*Ca.* Saccharimonadia") | 0 | 0 | 16S | Forward | GTT GGT AGT GCT CGC TGC AT | Batinovic *et al*., 2021 |
| Reverse primer for M. amalyticus | "*Ca.* Mycolatisynbacter gordoniilyticus" ("*Ca.* Saccharimonadia") | 0 | 0 | 16S | Reverse | TCA CCC GCC TCG TAT TGA CT | Batinovic *et al*., 2021 |
| 32-520-1066f | *Minisyncoccia* | 24 | 3 | 16S | Forward | GAG CAA CTC AAG CCA CCT GCT G | Kuroda *et al*., 2022B |
| AJ13-F | "*Ca.* Saccharimonadia" | 512 | 1 | 16S | Forward | GTG ACT GGG CGT AAA GAG TT | Nie *et al*., 2022 |
| AJ14-R | "*Ca.* Saccharimonadia" | 968 | 1777 | 16S | Reverse | CTA CGG ATT TCA CTC CTA C | Nie *et al*., 2022 |
| OD1_987f | *Minisyncoccia* (Specific for formerly "*Ca.* Nealsonbacteria") | 140 | 33442 | 16S | Forward | GGT GCT GCA TGG TTG TCG TC | Chen *et al*., 2023 |
| OD1_1186r | *Minisyncoccia* (Specific for formerly "*Ca.* Nealsonbacteria") | 1 | 15 | 16S | Reverse | GCT GCC CTC TGT AAC TGC CA | Chen *et al*., 2023 |
| 515F_Mod | Bacteria | N/E | N/E | 16S | Forward | GTG YCA GMA GBN KCG GTV A | Hu *et al*., 2024 |
| 806R_Mod | Bacteria | N/E | N/E | 16S | Reverse | RGA CTA MNV RGG THT CTA AT | Hu *et al*., 2024 |
| 684F-CPR | *Minisyncoccota* | 970* | 429* | 16S | Forward | GTA GKR RTR AAA TSC GTT | Man *et al*., 2024 |
| 1492r_CPR | *Minisyncoccota* | 51 | 0 | 16S | Reverse | TAC CCG TGC CTT GTT ACG ACT T | Nakajima *et al*., 2025 |

"Target" indicates the total number of sequences belonging to the target "Lineage" that show perfect matches (0 mismatches) to the primer. "Outgroup" indicates all non-target sequences in the dataset (i.e., total hit sequences minus "Target").

*Sequences containing ambiguous bases were evaluated in silico using the SILVA TestProbe tool (https://www.arb-silva.de/testprobe). Other sequences were evaluated using the ARB software v7.0 with SILVA_138.2_SSURef_NR99_03_07_24_opt.arb or SILVA_138.2_LSURef_NR99_03_07_24_opt.arb.

N/E: Not evaluated (not specific to *Minisyncoccota*).

## **Table S2**. List of FISH probes

| Probe name | Lineage | Target | Outgroup | SSU type | Probe type | Sequence (5'→3') | FA (%) | Reference |
|---|---|---|---|---|---|---|---|---|
| TM7305 | "*Ca.* Saccharimonadia" | 379 | 293 | 16S | Target | GTC CCA GTC TGG CTG ATC | 30 | Hugenholtz *et al.*, 2001 |
| TM7905 | "*Ca.* Saccharimonadia" | 1031 | 32 | 16S | Target | CCG TCA ATT CCT TTA TGT TTT A | 20 | Hugenholtz *et al.*, 2001 |
| TM7522 | "*Ca.* Saccharimonadia" | 1093 | 117 | 16S | Target | CGT ATG ACC GCG GCT G | N/A | Hugenholtz *et al.*, 2001 |
| TM7567 | "*Ca.* Saccharimonadia" | 988 | 25 | 16S | Target | CCT ACG CAA CTC TTT ACG CC | 30*[2] | Hugenholtz *et al.*, 2001 |
| I025-136 | "*Ca.* Saccharimonadia" | 4 | 0 | 16S | Target | GTC TTA TCC CTC ACT GCA GG | 20 | Brinig *et al.*, 2003 |
| DIGA11YD11 | WWE3 ("*Ca.* Katanibacteriota") | 5 | 0 | 16S | Target | TAG CAT TCA CCC TGA ACC | NA | Guermazi *et al.*, 2008 |
| TM7a-1033 | "*Ca.* Saccharimonadia" | 19 | 0 | 16S | Target | ATC TGT CAC CGA GTT CCA | 30 | Dinis *et al.*, 2011 |
| TM7.1010 | "*Ca.* Saccharimonadia" | 2 | 0 | 16S | Target | GGC ACA CGC TGC TTT CGC A | 35 | Albertsen *et al.*, 2013 |
| TM7.1010-c1 | - | - | - | 16S | Competitor | GGC ACA CTC TGC TTT CGC A | - | Albertsen *et al.*, 2013 |
| TM7.1010-c2 | - | - | - | 16S | Competitor | GGC ACA CGT TGC TTT CGC A | - | Albertsen *et al.*, 2013 |
| TM7.1010-h989 | - | - | - | 16S | Helper | GTG CTT CCC TGG ATG TCA AGC | - | Albertsen *et al.*, 2013 |
| TM7.1010-h1029 | - | - | - | 16S | Helper | CAA CAC CTG TCA CAG GGT TCC AAA A | - | Albertsen *et al.*, 2013 |
| TM7.94 | "*Ca.* Saccharimonadia" | 1 | 0 | 16S | Target | CAC TCG ACA GCA CTT TCA CTC | 35 | Albertsen *et al.*, 2013 |
| TM7.94-c | - | - | - | 16S | Competitor | CAC TCG TCA GC ACT TTC ACT C | - | Albertsen *et al.*, 2013 |
| TM7.94-h83 | - | - | - | 16S | Helper | TTC ACT CCT CGA TAT TCT ATC GAA T | - | Albertsen *et al.*, 2013 |
| TM7.94-h105 | - | - | - | 16S | Helper | CGT TAC TCA GCC GTC CGC | - | Albertsen *et al.*, 2013 |
| TM7.584 | "*Ca.* Saccharimonadia" | 2 | 0 | 16S | Target | ATT CGC TTA TAC GAC CAC C | 35 | Albertsen *et al.*, 2013 |
| TM7.584-c1 | - | - | - | 16S | Competitor | ATT CGC TTA TAA GAC CAC C | - | Albertsen *et al.*, 2013 |
| TM7.584-c2 | - | - | - | 16S | Competitor | ATT TGC TTA TAC GAC CAC C | - | Albertsen *et al.*, 2013 |
| TM7.584-c3 | - | - | - | 16S | Competitor | ATT CAC TTA TAC AAC CAC C | - | Albertsen *et al.*, 2013 |
| TM7.584-h565 | - | - | - | 16S | Helper | TAC GCA ACT CTT TAC GCC CA | - | Albertsen *et al.*, 2013 |
| TM7.584-h603 | - | - | - | 16S | Helper | TTG AGC CAC CAG ATT TCA CT | - | Albertsen *et al.*, 2013 |
| TM7-305 Competitor probe A | - | - | - | 16S | Competitor | GTC CCA GTC TGG CTG -TC | - | Nittami *et al.*, 2014 |
| TM7-305 Competitor probe B | - | - | - | 16S | Competitor | GTC CCA GTC TGG CTG GTT | - | Nittami *et al.*, 2014 |
| TM7-905 Competitor probe | - | - | - | 16S | Competitor | CCG TCA ATT CCT TTA TGT TTT A | - | Nittami *et al.*, 2014 |
| BD1207 | "*Ca.* Gracilibacteria" | 115 | 2 | 16S | Target | AGC CCC AGA CGT AAA AGC | 40 | Hanke *et al.*, 2014 |
| OD1-613 | *Minisyncoccia* | 2 | 0 | 16S | Target | GGG CCT GAA GTT GAG CTC C | 30 | Gong *et al.*, 2014 |
| OD1-289 | *Minisyncoccia* | 172 | 1 | 16S | Target | GTC AGG CTC TCA CCT CCC | 30 | Gong *et al.*, 2014 |
| OD1-290 | *Minisyncoccia* | 172 | 1 | 16S | Target | GGT CAG GCT CTC ACC TCC | 20 | Gong *et al.*, 2014 |
| G3-specific probe | "*Ca.* Saccharimonadia" | 4 | 0 | 16S | Target | ACT TGG CTA TTA TGC GAG T | 30(42°C) | McLean *et al.*, 2020 |
| G5-specific probe | "*Ca.* Saccharimonadia" | 5 | 0 | 16S | Target | GCT CTT CGG AGT ACA CGA GA | 30(42°C) | McLean *et al.*, 2020 |
| G6-specific probe | "*Ca.* Saccharimonadia" | 18 | 126 | 16S | Target | GCA TCG AAA GGT GTG C | 30(42°C) | McLean *et al.*, 2020 |
| SR1-specific probe | "*Ca.* Gracilibacteria" | 163*[1] | 6*[1] | 16S | Target | TTA ACY RGA CAC CTT GCG | 30(42°C) | McLean *et al.*, 2020 |
| Pac683 | *Minisyncoccia* | 445 | 65 | 16S | Target | TCA ACG GAT TTC ACC CCT ACA C | 25 | Singleton *et al.*, 2021 |
| SacchariP | "*Ca.* Saccharimonadia" | 2 | 7 | 23S | Target | CAT AGA CGG CGC TGT TTG GCA C | - | Ibrahim *et al.*, 2021 |
| ABY1a-193 | "*Ca.* Patescibacteriia" | 1 | 0 | 16S | Target | AAG CGC CGA AGC TTT ACA AGG GAA TGT C | 40-45 | Chiriac *et al.*, 2022 |
| ABY1b-1343 | "*Ca.* Patescibacteriia" | 20*[1] | 1*[1] | 16S | Target | CGT DAT GAT CYR CCT TTA CTA GC | 45-50 | Chiriac *et al.*, 2022 |
| ABY1b-C1 | - | - | - | 16S | Competitor | CGT GAT GAT CCG CgA TTA CTA GC | - | Chiriac *et al.*, 2022 |
| ABY1b-C2 | - | - | - | 16S | Competitor | CGT GAT GAT gCG CCA TTA CTA GC | - | Chiriac *et al.*, 2022 |
| ABY1b-C3 | - | - | - | 16S | Competitor | CGT GcT GAT CCG CsA TTA CTA GC | - | Chiriac *et al.*, 2022 |
| ABY1b-C4 | - | - | - | 16S | Competitor | CGT GcT GAT CCG Cak TTA CTA GC | - | Chiriac *et al.*, 2022 |
| adl1-132 | *Minisyncoccia* | 2*[1] | 0*[1] | 16S | Target | AGC TAT ACC RGA CTY TTC GGT GC | 55-65 | Chiriac *et al.*, 2022 |
| adl2-134 | *Minisyncoccia* | 1 | 0 | 16S | Target | GCT ATG CCC GAC TTC TCG GT | 55-65 | Chiriac *et al.*, 2022 |
| pgri-99 | "*Ca.* Gracilibacteria" | 3 | 0 | 16S | Target | CTC AGC CGT CCG CCG TGG G | 70 | Chiriac *et al.*, 2022 |
| pgri-124 | "*Ca.* Gracilibacteria" | 1 | 0 | 16S | Target | CCT GAG TTC AAG GCA GGT ACC AAC | 55-65 | Chiriac *et al.*, 2022 |
| pgri-124-C1 | - | - | - | 16S | Competitor | CgC AGC CGT CCG CCG TGG G | - | Chiriac *et al.*, 2022 |
| pgri-124-C2 | - | - | - | 16S | Competitor | CTC AGC CGT CtG CCG TGG G | - | Chiriac *et al.*, 2022 |
| SacA-77 | "*Ca.* Saccharimonadia" | 0 | 0 | 16S | Target | TGT CCG AAG ACT GAC TTG TCT GGT CAA | 40-45 | Chiriac *et al.*, 2022 |
| ZE-1429 | *Minisyncoccia* | 0 | 0 | 16S | Target | TCC CAC ACA TGG TGA GAT ATC GGG | 60-65 | Chiriac *et al.*, 2022 |
| 32-520-1066 | *Minisyncoccia* | 24 | 3 | 16S | Target | GAG CAA CTC AAG CCA CCT GCT G | 25 | Kuroda *et al.*, 2022B |
| GRA665 | "*Ca.* Gracilibacteria" | 1 | 0 | 16S | Target | TTA CCG TTC TGC TAG CCC | 30 | Fujii *et al.*, 2024 |
| GRA686 | "*Ca.* Gracilibacteria" | 16 | 0 | 16S | Target | CAA CGG ATT GCA CCC CTA | 30 | Fujii *et al.*, 2024 |
| CompGRA686 | - | - | - | 16S | Competitor | CAA CGG ATT TCA CCC CTA | - | Fujii *et al.*, 2024 |

“Target” indicates the total number of sequences belonging to the target “Lineage” that show perfect matches (0 mismatches) to the FISH probe. “Outgroup” indicates all non-target sequences in the dataset (i.e., total hit sequences minus “Target”).

*[1]Sequences containing ambiguous bases were evaluated in silico using the SILVA TestProbe tool (https://www.arb-silva.de/testprobe). Other sequences were evaluated using the ARB software v7.0 with SILVA_138.2_SSURef_NR99_03_07_24_opt.arb or SILVA_138.2_LSURef_NR99_03_07_24_opt.arb.

*[2]Not mentioned in the original publication but described in Kagemasa *et al*. (2022).

## **Table S3.** Microorganisms forming parasitic or predatory associations with the phylum *Minisyncoccota.*

| *Minisyncoccota* | | | Host microorganism | | | | | |
|---|---|---|---|---|---|---|---|---|
| Class | Species | Strain | Domain | Phylum | Species | Strain | Environment | Reference |
| *Minisyncoccia* | “*Ca.* Sonnebornia yantaiensis” | - | Eukaryota | *Ciliophora* | *Paramecium bursaria* | - | Pond | Gong *et al.*, 2014 |
| “*Ca.* Saccharimonadia” | “*Ca.* Nanosynbacter lyticus” | TM7x | Bacteria | *Actinomycetota* | *Schaalia odontolytica* | XH001 | Oral | He *et al.*, 2015 Bor *et al.*, 2016, 2018, Utter *et al.*, 2020 |
| “*Ca.* Saccharimonadia” | “*Ca.* Nanosynbacter lyticus” | TM7x | Bacteria | *Actinomycetota* | *Schaalia odontolytica* | ATCC17929 | Oral | Utter *et al.*, 2020 |
| “*Ca.* Saccharimonadia” | “*Ca.* Nanosynbacter lyticus” | TM7x | Bacteria | *Actinomycetota* | *Schaalia odontolytica* | ATCC17982 | Oral | Bor *et al.*, 2018; Utter *et al.*, 2020 |
| “*Ca.* Saccharimonadia” | “*Ca.* Nanosynbacter lyticus” | TM7x | Bacteria | *Actinomycetota* | *Schaalia odontolytica* | F0309 | Oral | Utter *et al.*, 2020 |
| “*Ca.* Saccharimonadia” | “*Ca.* Nanosynbacter lyticus” | TM7x | Bacteria | *Actinomycetota* | *Schaalia meyeri* | W712 | Oral | Bor *et al.*, 2018; Utter *et al.*, 2020 |
| “*Ca.* Saccharimonadia” | “*Ca.* Nanosynbacter lyticus” | TM7x | Bacteria | *Actinomycetota* | *Schaalia meyeri* | ATCC35568 | Oral | Utter *et al.*, 2020 |
| “*Ca.* Saccharimonadia” | “*Ca.* Nanosynbacter lyticus” | TM7x | Bacteria | *Actinomycetota* | *Actinomyces* sp. | F0543 | Oral | Bor *et al.*, 2018; Utter *et al.*, 2020 |
| “*Ca.* Saccharimonadia” | “*Ca.* Nanosynbacter lyticus” | TM7x | Bacteria | *Actinomycetota* | *Actinomyces* sp. | F0338 | Oral | Bor *et al.*, 2018; Utter *et al.*, 2020 |
| “*Ca.* Saccharimonadia” | “*Ca.* Nanosynbacter lyticus” | TM7x | Bacteria | *Actinomycetota* | *Actinomyces* sp. | F0310 | Oral | Utter *et al.*, 2020 |
| “*Ca.* Saccharimonadia” | “*Ca.* Nanosynbacter lyticus” | TM7x | Bacteria | *Actinomycetota* | *Actinomyces* sp. | ICM58 | Oral | Bor *et al.*, 2018; Utter *et al.*, 2020 |
| “*Ca.* Saccharimonadia” | “*Ca.* Nanosynbacter lyticus” | TM7x | Bacteria | *Actinomycetota* | *Actinomyces* sp. | F0311 | Oral | Bor *et al.*, 2018; Utter *et al.*, 2020 |
| “*Ca.* Saccharimonadia” | “*Ca.* Nanosynbacter lyticus” | TM7x | Bacteria | *Actinomycetota* | *Actinomyces* sp. | ICM47 | Oral | Bor *et al.*, 2018; Utter *et al.*, 2020 |
| “*Ca.* Saccharimonadia” | “*Ca.* Nanosynbacter lyticus” | TM7x | Bacteria | *Actinomycetota* | *Actinomyces* sp. | ICM39 | Oral | Bor *et al.*, 2018; Utter *et al.*, 2020 |
| “*Ca.* Saccharimonadia” | “*Ca.* Nanosynbacter” sp. | TM7_HOT_352-SAGs1-8 | Bacteria | *Actinomycetota* | *Schaalia odontolytica* | OR | Oral | Cross *et al.*, 2019 |
| “*Ca.* Saccharimonadia” | “*Ca.* Nanosynbacter” sp. | TM7_HOT_351-SAGs1-4 | Bacteria | *Actinomycetota* | *Actinomyces* sp. | N/A | Oral | Cross *et al.*, 2019 |
| “*Ca.* Saccharimonadia” | “*Ca.* Nanosynbacter” sp. | TM7_HOT_346-SAGs1-3 | Bacteria | *Actinomycetota* | *Actinomyces* sp. | N/A | Oral | Cross *et al.*, 2019 |
| “*Ca.* Saccharimonadia” | “*Ca.* Nanosynbacter” sp. | TM7_HOT_346-SAGs1-3 | Bacteria | *Actinomycetota* | *Cellulosimicrobium celullans* | N/A | Oral | Cross *et al.*, 2019 |
| “*Ca.* Saccharimonadia” | “*Ca.* Nanosynbacter” sp. | TM7_HOT_348-SAGs1-4 | Bacteria | *Actinomycetota* | *Cellulosimicrobium celullans* | N/A | Oral | Cross *et al.*, 2019 |
| “*Ca.* Saccharimonadia” | “*Ca.* Nanosynbacter featherlites”[*1] | BB001 | Bacteria | *Actinomycetota* | *Actinomyces* sp. | F0337 | Oral | Bor *et al.*, 2020 |
| “*Ca.* Saccharimonadia” | “*Ca.* Nanosynbacter featherlites”[*1] | BB001 | Bacteria | *Actinomycetota* | *Actinomyces massiliensis* | F0489 | Oral | Bor *et al.*, 2020 |
| “*Ca.* Saccharimonadia” | “*Ca.* Nanosynbacter” sp. | AC001 | Bacteria | *Actinomycetota* | *Arachnia propionica* | F0230 | Oral | Bor *et al.*, 2020 |
| “*Ca.* Saccharimonadia” | “*Ca.* Nanosynbacter” sp. | AC001 | Bacteria | *Actinomycetota* | *Arachnia propionica* | F0700 | Oral | Bor *et al.*, 2020; Murugkar *et al.*, 2020 |
| “*Ca.* Saccharimonadia” | “*Ca.* Saccharimonas” sp. | PM004 | Bacteria | *Actinomycetota* | *Arachnia propionica* | F0230 | Oral | Bor *et al.*, 2020 |
| “*Ca.* Saccharimonadia” | “*Ca.* Saccharimonas” sp. | PM004 | Bacteria | *Actinomycetota* | *Arachnia propionica* | F0700 | Oral | Bor *et al.*, 2020; Murugkar *et al.*, 2020 |
| “*Ca.* Saccharimonadia” | “*Ca.* Nanosynbacter” sp. | CM001 | Bacteria | *Actinomycetota* | *Arachnia propionica* | F0700 | Oral | Murugkar *et al.*, 2020 |
| “*Ca.* Saccharimonadia” | “*Ca.* Nanosynbacter” sp. | CM002 | Bacteria | *Actinomycetota* | *Arachnia propionica* | F0700 | Oral | Murugkar *et al.*, 2020 |
| “*Ca.* Saccharimonadia” | “*Ca.* Saccharimonas” sp. | CM003 | Bacteria | *Actinomycetota* | *Arachnia propionica* | F0700 | Oral | Murugkar *et al.*, 2020 |
| “*Ca.* Saccharimonadia” | “*Ca.* Saccharimonas” sp. | CM004 | Bacteria | *Actinomycetota* | *Arachnia propionica* | F0700 | Oral | Murugkar *et al.*, 2020 |
| “*Ca.* Saccharimonadia” | “*Ca.* Nanosynbacter” sp. | CM005 | Bacteria | *Actinomycetota* | *Arachnia propionica* | F0700 | Oral | Murugkar *et al.*, 2020 |
| “*Ca.* Saccharimonadia” | “*Ca.* Nanosynbacter” sp. | CM006 | Bacteria | *Actinomycetota* | *Arachnia propionica* | F0700 | Oral | Murugkar *et al.*, 2020 |
| “*Ca.* Saccharimonadia” | “*Ca.* Nanosynbacter” sp. | CM007 | Bacteria | *Actinomycetota* | *Arachnia propionica* | F0700 | Oral | Murugkar *et al.*, 2020 |
| “*Ca.* Saccharimonadia” | “*Ca.* Nanosynbacter” sp. | CM008 | Bacteria | *Actinomycetota* | *Arachnia propionica* | F0700 | Oral | Murugkar *et al.*, 2020 |
| “*Ca.* Saccharimonadia” | “*Ca.* Saccharimonas” sp. | CM009 | Bacteria | *Actinomycetota* | *Arachnia propionica* | F0700 | Oral | Murugkar *et al.*, 2020 |
| “*Ca.* Saccharimonadia” | “*Ca.* Nanosynbacter” sp. | CM010 | Bacteria | *Actinomycetota* | *Arachnia propionica* | F0700 | Oral | Murugkar *et al.*, 2020 |
| “*Ca.* Saccharimonadia” | “*Ca.* Nanosynbacter” sp. | FS03P | Bacteria | *Actinomycetota* | *Arachnia propionica* | F0700 | Oral | Murugkar *et al.*, 2020 |
| “*Ca.* Saccharimonadia” | “*Ca.* Nanosynbacter” sp. | FS05P-B | Bacteria | *Actinomycetota* | *Arachnia propionica* | F0700 | Oral | Murugkar *et al.*, 2020 |
| “*Ca.* Saccharimonadia” | “*Ca.* Nanosynbacter” sp. | FS07P | Bacteria | *Actinomycetota* | *Arachnia propionica* | F0700 | Oral | Murugkar *et al.*, 2020 |
| “*Ca.* Saccharimonadia” | “*Ca.* Nanosynbacter” sp. | FS013P | Bacteria | *Actinomycetota* | *Arachnia propionica* | F0700 | Oral | Murugkar *et al.*, 2020 |
| “*Ca.* Saccharimonadia” | “*Ca.* Saccharimonas” sp. | FS014P | Bacteria | *Actinomycetota* | *Arachnia propionica* | F0700 | Oral | Murugkar *et al.*, 2020 |
| “*Ca.* Saccharimonadia” | “*Ca.* Nanosynbacter” sp. | FS015P | Bacteria | *Actinomycetota* | *Arachnia propionica* | F0700 | Oral | Murugkar *et al.*, 2020 |
| “*Ca.* Saccharimonadia” | “*Ca.* Saccharimonas” sp. | FS017P | Bacteria | *Actinomycetota* | *Arachnia propionica* | F0700 | Oral | Murugkar *et al.*, 2020 |
| “*Ca.* Saccharimonadia” | “*Ca.* Saccharimonas” sp. | AC002 | Bacteria | *Actinomycetota* | *Arachnia propionica* | F0700 | Oral | Murugkar *et al.*, 2020 |
| “*Ca.* Saccharimonadia” | “*Ca.* Nanosynbacter” sp. | FS04P | Bacteria | *Actinomycetota* | *Arachnia propionica* | F0700 | Oral | Murugkar *et al.*, 2020 |
| “*Ca.* Saccharimonadia” | “*Ca.* Saccharimonas” sp. | FS05P-A | Bacteria | *Actinomycetota* | *Arachnia propionica* | F0700 | Oral | Murugkar *et al.*, 2020 |
| “*Ca.* Saccharimonadia” | “*Ca.* Saccharimonas” sp. | FS06P-A | Bacteria | *Actinomycetota* | *Arachnia propionica* | F0700 | Oral | Murugkar *et al.*, 2020 |
| “*Ca.* Saccharimonadia” | “*Ca.* Saccharimonas” sp. | FS06P-B | Bacteria | *Actinomycetota* | *Arachnia propionica* | F0700 | Oral | Murugkar *et al.*, 2020 |
| “*Ca.* Saccharimonadia” | “*Ca.* Nanosynbacter” sp. | FS09P | Bacteria | *Actinomycetota* | *Arachnia propionica* | F0700 | Oral | Murugkar *et al.*, 2020 |
| “*Ca.* Saccharimonadia” | “*Ca.* Saccharimonas” sp. | HB001 | Bacteria | *Actinomycetota* | *Arachnia propionica* | F0230 | Oral | Murugkar *et al.*, 2020 |

**Table S3.** Continued.

| *Minisyncoccota* | | | Host microorganism | | | | | |
|---|---|---|---|---|---|---|---|---|
| Class | Species | Strain | Domain | Phylum | Species | Strain | Environment | Reference |
| "*Ca.* Saccharimonadia" | "*Ca.* Nanosynbacter lyticus" | FS04A | Bacteria | *Actinomycetota* | *Schaalia meyeri* | W712 | Oral | Bor *et al*., 2018; Utter *et al*., 2020; Murugkar *et al*., 2020 |
| "*Ca.* Saccharimonadia" | "*Ca.* Nanosynbacter lyticus" | FS06A | Bacteria | *Actinomycetota* | *Schaalia meyeri* | W712 | Oral | Bor *et al*., 2018; Utter *et al*., 2020; Murugkar *et al*., 2020 |
| "*Ca.* Saccharimonadia" | "*Ca.* Nanosynbacter lyticus" | FS07A | Bacteria | *Actinomycetota* | *Schaalia meyeri* | W712 | Oral | Bor *et al*., 2018; Utter *et al*., 2020; Murugkar *et al*., 2020 |
| "*Ca.* Saccharimonadia" | "*Ca.* Nanosynbacter lyticus" | FS09A | Bacteria | *Actinomycetota* | *Schaalia meyeri* | W712 | Oral | Bor *et al*., 2018; Utter *et al*., 2020; Murugkar *et al*., 2020 |
| "*Ca.* Saccharimonadia" | "*Ca.* Nanosynbacter lyticus" | FS10A | Bacteria | *Actinomycetota* | *Schaalia meyeri* | W712 | Oral | Bor *et al*., 2018; Utter *et al*., 2020; Murugkar *et al*., 2020 |
| "*Ca.* Saccharimonadia" | "*Ca.* Saccharimonas" sp. | PM007 | Bacteria | *Actinomycetota* | *Actinomyces* sp. | PM007 | Oral | Murugkar *et al*., 2020 |
| "*Ca.* Gracilibacteria" | "*Ca.* Vampirococcus lugosii" | Chiprana | Bacteria | *Pseudomonadota* | *Halochromatium* sp. | - | lake | Moreira *et al*., 2021 |
| "*Ca.* Saccharimonadia" | "*Ca.* Nanosynbacter" sp. | BB002 | Bacteria | *Actinomycetota* | *Actinomyces* sp. | F0337 | Oral | Chipashvili *et al*., 2021 |
| "*Ca.* Saccharimonadia" | "*Ca.* Nanosynbacter" sp. | BB003 | Bacteria | *Actinomycetota* | *Schaalia meyeri* | W712 | Oral | Chipashvili *et al*., 2021 |
| "*Ca.* Saccharimonadia" | "*Ca.* Nanosynbacter" sp. | BB004 | Bacteria | *Actinomycetota* | *Schaalia meyeri* | W712 | Oral | Chipashvili *et al*., 2021 |
| "*Ca.* Saccharimonadia" | "*Ca.* Saccharimonas" sp. | BB006 | Bacteria | *Actinomycetota* | *Arachnia propionica* | F0700 | Oral | Chipashvili *et al*., 2021 |
| "*Ca.* Saccharimonadia" | "*Ca.* Nanosynbacter" sp. | BB008 | Bacteria | *Actinomycetota* | *Arachnia propionica* | F0700 | Oral | Chipashvili *et al*., 2021 |
| "*Ca.* Saccharimonadia" | "*Ca.* Mycolatisynbacter gordoniilyticus" | JR1 | Bacteria | *Actinomycetota* | *Dietzia maris* | CON27 | Lab [*2] | Batinovic *et al*., 2021 |
| "*Ca.* Saccharimonadia" | "*Ca.* Mycolatisynbacter gordoniilyticus" | JR1 | Bacteria | *Actinomycetota* | *Gordonia alkanivorans* | CON72 | Lab [*2] | Batinovic *et al*., 2021 |
| "*Ca.* Saccharimonadia" | "*Ca.* Mycolatisynbacter gordoniilyticus" | JR1 | Bacteria | *Actinomycetota* | *Gordonia amarae* | CON44 | Activated sludge [*2] | Batinovic *et al*., 2021 |
| "*Ca.* Saccharimonadia" | "*Ca.* Mycolatisynbacter gordoniilyticus" | JR1 | Bacteria | *Actinomycetota* | *Gordonia amarae* | BEN368 | Activated sludge [*2] | Batinovic *et al*., 2021 |
| "*Ca.* Saccharimonadia" | "*Ca.* Mycolatisynbacter gordoniilyticus" | JR1 | Bacteria | *Actinomycetota* | *Gordonia amarae* | BEN372 | Activated sludge [*2] | Batinovic *et al*., 2021 |
| "*Ca.* Saccharimonadia" | "*Ca.* Mycolatisynbacter gordoniilyticus" | JR1 | Bacteria | *Actinomycetota* | *Gordonia amarae* | BEN374 | Activated sludge [*2] | Batinovic *et al*., 2021 |
| "*Ca.* Saccharimonadia" | "*Ca.* Mycolatisynbacter gordoniilyticus" | JR1 | Bacteria | *Actinomycetota* | *Gordonia defluvii* | J5 | Lab [*2] | Batinovic *et al*., 2021 |
| "*Ca.* Saccharimonadia" | "*Ca.* Mycolatisynbacter gordoniilyticus" | JR1 | Bacteria | *Actinomycetota* | *Gordonia desulfuricans* | CON69 | Lab [*2] | Batinovic *et al*., 2021 |
| "*Ca.* Saccharimonadia" | "*Ca.* Mycolatisynbacter gordoniilyticus" | JR1 | Bacteria | *Actinomycetota* | *Gordonia malaquae* | N/A | Lab [*2] | Batinovic *et al*., 2021 |
| "*Ca.* Saccharimonadia" | "*Ca.* Mycolatisynbacter gordoniilyticus" | JR1 | Bacteria | *Actinomycetota* | *Gordonia obuensis* | CON31 | Lab [*2] | Batinovic *et al*., 2021 |
| "*Ca.* Saccharimonadia" | "*Ca.* Mycolatisynbacter gordoniilyticus" | JR1 | Bacteria | *Actinomycetota* | *Gordonia pseudoamarae* | CON9 | Activated sludge [*2] | Batinovic *et al*., 2021 |
| "*Ca.* Saccharimonadia" | "*Ca.* Mycolatisynbacter gordoniilyticus" | JR1 | Bacteria | *Actinomycetota* | *Gordonia pseudoamarae* | BEN371 | Activated sludge [*2] | Batinovic *et al*., 2021 |
| "*Ca.* Saccharimonadia" | "*Ca.* Mycolatisynbacter gordoniilyticus" | JR1 | Bacteria | *Actinomycetota* | *Gordonia rubripertincta* | CON38 | Lab [*2] | Batinovic *et al*., 2021 |
| "*Ca.* Saccharimonadia" | "*Ca.* Mycolatisynbacter gordoniilyticus" | JR1 | Bacteria | *Actinomycetota* | *Gordonia sputi* | CON48 | Lab [*2] | Batinovic *et al*., 2021 |
| "*Ca.* Saccharimonadia" | "*Ca.* Mycolatisynbacter gordoniilyticus" | JR1 | Bacteria | *Actinomycetota* | *Gordonia terrae* | CON34 | Lab [*2] | Batinovic *et al*., 2021 |
| "*Ca.* Saccharimonadia" | "*Ca.* Mycolatisynbacter gordoniilyticus" | JR1 | Bacteria | *Actinomycetota* | *Millisia brevis* | J82 | Lab [*2] | Batinovic *et al*., 2021 |
| "*Ca.* Saccharimonadia" | "*Ca.* Mycolatisynbacter gordoniilyticus" | JR1 | Bacteria | *Actinomycetota* | *Nocardia asteroides* | CON20 | Lab [*2] | Batinovic *et al*., 2021 |
| "*Ca.* Saccharimonadia" | "*Ca.* Mycolatisynbacter gordoniilyticus" | JR1 | Bacteria | *Actinomycetota* | *Nocardia brasiliensis* | CON42 | Lab [*2] | Batinovic *et al*., 2021 |
| "*Ca.* Saccharimonadia" | "*Ca.* Mycolatisynbacter gordoniilyticus" | JR1 | Bacteria | *Actinomycetota* | *Nocardia brevicatena* | CON43 | Lab [*2] | Batinovic *et al*., 2021 |
| "*Ca.* Saccharimonadia" | "*Ca.* Mycolatisynbacter gordoniilyticus" | JR1 | Bacteria | *Actinomycetota* | *Nocardia carnea* | CON30 | Lab [*2] | Batinovic *et al*., 2021 |
| "*Ca.* Saccharimonadia" | "*Ca.* Mycolatisynbacter gordoniilyticus" | JR1 | Bacteria | *Actinomycetota* | *Nocardia erythropolis* | CON19 | Lab [*2] | Batinovic *et al*., 2021 |
| "*Ca.* Saccharimonadia" | "*Ca.* Mycolatisynbacter gordoniilyticus" | JR1 | Bacteria | *Actinomycetota* | *Nocardia otitidiscaviarum* | CON25 | Lab [*2] | Batinovic *et al*., 2021 |
| "*Ca.* Saccharimonadia" | "*Ca.* Mycolatisynbacter gordoniilyticus" | JR1 | Bacteria | *Actinomycetota* | *Nocardia transvalensis* | CON40 | Lab [*2] | Batinovic *et al*., 2021 |
| "*Ca.* Saccharimonadia" | "*Ca.* Mycolatisynbacter gordoniilyticus" | JR1 | Bacteria | *Actinomycetota* | *Rhodococcus aichiensis* | CON22 | Lab [*2] | Batinovic *et al*., 2021 |
| "*Ca.* Saccharimonadia" | "*Ca.* Mycolatisynbacter gordoniilyticus" | JR1 | Bacteria | *Actinomycetota* | *Rhodococcus australis* | N/A | Lab [*2] | Batinovic *et al*., 2021 |
| "*Ca.* Saccharimonadia" | "*Ca.* Mycolatisynbacter gordoniilyticus" | JR1 | Bacteria | *Actinomycetota* | *Rhodococcus coprophilus* | CON18 | Lab [*2] | Batinovic *et al*., 2021 |
| "*Ca.* Saccharimonadia" | "*Ca.* Mycolatisynbacter gordoniilyticus" | JR1 | Bacteria | *Actinomycetota* | *Rhodococcus equi* | CON10 | Lab [*2] | Batinovic *et al*., 2021 |
| "*Ca.* Saccharimonadia" | "*Ca.* Mycolatisynbacter gordoniilyticus" | JR1 | Bacteria | *Actinomycetota* | *Rhodococcus erythropolis* | CON29 | Lab [*2] | Batinovic *et al*., 2021 |

## **Table S3.** Continued.

| *Minisyncoccota* | | | Host microorganism | | | | | |
|---|---|---|---|---|---|---|---|---|
| Class | Species | Strain | Domain | Phylum | Species | Strain | Environment | Reference |
| "*Ca.* Saccharimonadia" | "*Ca.* Mycolatisynbacter gordoniilyticus" | JR1 | Bacteria | *Actinomycetota* | *Rhodococcus globerulus* | CON35 | Lab [*2] | Batinovic *et al.*, 2021 |
| "*Ca.* Saccharimonadia" | "*Ca.* Mycolatisynbacter gordoniilyticus" | JR1 | Bacteria | *Actinomycetota* | *Rhodococcus rhodnii* | CON46 | Lab [*2] | Batinovic *et al.*, 2021 |
| "*Ca.* Saccharimonadia" | "*Ca.* Mycolatisynbacter gordoniilyticus" | JR1 | Bacteria | *Actinomycetota* | *Rhodococcus rhodochrous* | CON11 | Lab [*2] | Batinovic *et al.*, 2021 |
| "*Ca.* Saccharimonadia" | "*Ca.* Mycolatisynbacter gordoniilyticus" | JR1 | Bacteria | *Actinomycetota* | *Rhodococcus ruber* | CON33 | Lab [*2] | Batinovic *et al.*, 2021 |
| "*Ca.* Saccharimonadia" | "*Ca.* Mycolatisynbacter gordoniilyticus" | JR1 | Bacteria | *Actinomycetota* | *Rhodococcus triatomae* | RHO1 | Lab [*2] | Batinovic *et al.*, 2021 |
| "*Ca.* Saccharimonadia" | "*Ca.* Nanosynbacter vallesae"[*1] | IHU2 | Bacteria | *Actinomycetota* | *Schaalia odontolytica* | N/A | Oral | Ibrahim *et al.*, 2021 |
| "*Ca.* Saccharimonadia" | "*Ca.* Nanosynbacter" sp. | IHU1 | Bacteria | *Actinomycetota* | *Schaalia odontolytica* | N/A | Oral | Ibrahim *et al.*, 2021 |
| *Minisyncoccia* | "*Ca.* Yanofskyibacterium parasiticum" | PMX.810_sub | Archaea | *Halobacteriota* | *Methanothrix soehngenii* | GP6 | Lab. reactor | Kuroda *et al.*, 2022A; Kuroda *et al.*, 2024 |
| *Minisyncoccia* | *Minisyncoccus archaeiphilus* | PMX.108 | Archaea | *Halobacteriota* | *Methanospirillum hungatei* | DSM 864 | Lab. reactor | Kuroda *et al.*, 2022 B; Kuroda *et al.*, 2024; Nakajima *et al.*, 2025 |
| *Minisyncoccia* | "*Ca.* Microsyncoccus archaeolyticus" | PMX.50 | Archaea | *Halobacteriota* | *Methanospirillum* sp. | N/A | Lab. reactor | Kuroda *et al.*, 2022 B; Kuroda *et al.*, 2024 |
| "*Ca.* Saccharimonadia" | "*Ca.* Microsaccharimonas" sp. | TM7i | Bacteria | *Actinomycetota* | *Leucobacter aridicollis* | J1 | Cicadae Periostracum | Xie *et al.*, 2022 |
| "*Ca.* Gracilibacteria" | "*Ca.* Absconditicoccus praedator" | M39-6 | Bacteria | *Pseudomonadota* | *Halorhodospira halophila* | M39-5 | Lake | Yakimov *et al.*, 2022 |
| "*Ca.* Saccharimonadia" | "*Ca.* Nanosynbacter" sp. | TM7-001 | Bacteria | *Actinomycetota* | *Schaalia meyeri* | W712 | Oral | Nie *et al.*, 2022 |
| "*Ca.* Saccharimonadia" | "*Ca.* Nanosynbacter" sp. | TM7-008 | Bacteria | *Actinomycetota* | *Schaalia meyeri* | W712 | Oral | Nie *et al.*, 2022 |
| "*Ca.* Saccharimonadia" | "*Ca.* Nanosynbacter" sp. | TM7-033 | Bacteria | *Actinomycetota* | *Actinomyces* sp. | JN023 | Oral | Nie *et al.*, 2022 |
| "*Ca.* Saccharimonadia" | "*Ca.* Nanosynbacter" sp. | TM7-037 | Bacteria | *Actinomycetota* | *Schaalia cardiffensis* | F0333 | Oral | Nie *et al.*, 2022 |
| "*Ca.* Saccharimonadia" | "*Ca.* Nanosynbacter" sp. | TM7-053 | Bacteria | *Actinomycetota* | *Actinomyces* sp. | JN014 | Oral | Nie *et al.*, 2022 |
| "*Ca.* Saccharimonadia" | "*Ca.* Nanosynbacter" sp. | TM7-057 | Bacteria | *Actinomycetota* | *Actinomyces* sp. | JN023 | Oral | Nie *et al.*, 2022 |
| "*Ca.* Saccharimonadia" | "*Ca.* Nanosynbacter" sp. | TM7-072 | Bacteria | *Actinomycetota* | *Actinomyces* sp. | ICM47 | Oral | Nie *et al.*, 2022 |
| "*Ca.* Saccharimonadia" | "*Ca.* Nanosynbacter" sp. | TM7-075 | Bacteria | *Actinomycetota* | *Actinomyces* sp. | JN023 | Oral | Nie *et al.*, 2022 |
| "*Ca.* Saccharimonadia" | "*Ca.* Nanosynbacter" sp. | TM7-076 | Bacteria | *Actinomycetota* | *Schaalia meyeri* | W712 | Oral | Nie *et al.*, 2022 |
| "*Ca.* Saccharimonadia" | "*Ca.* Nanosynbacter" sp. | TM7-087 | Bacteria | *Actinomycetota* | *Schaalia cardiffensis* | F0333 | Oral | Nie *et al.*, 2022 |
| "*Ca.* Saccharimonadia" | "*Ca.* Nanosynbacter" sp. | TM7-079 | Bacteria | *Actinomycetota* | *Schaalia meyeri* | W712 | Oral | Nie *et al.*, 2022 |
| "*Ca.* Saccharimonadia" | "*Ca.* Nanosynbacter" sp. | TM7-065 | Bacteria | *Actinomycetota* | *Actinomyces* sp. | F0337 | Oral | Nie *et al.*, 2022 |
| "*Ca.* Saccharimonadia" | "*Ca.* Nanosynbacter" sp. | TM7-069 | Bacteria | *Actinomycetota* | *Schaalia meyeri* | ATCC355 68 | Oral | Nie *et al.*, 2022 |
| "*Ca.* Saccharimonadia" | "*Ca.* Nanosynbacter" sp. | TM7-074 | Bacteria | *Actinomycetota* | *Schaalia meyeri* | W712 | Oral | Nie *et al.*, 2022 |
| *Minisyncoccia* | UBA5738 sp. [*3] | DGGOD1a | Archaea | *Halobacteriota* | *Methanothrix* sp. | N/A | Lab reactor | Chen *et al.*, 2023 |
| "*Ca.* Gracilibacteria" | JAGOMW01 sp. [*3] | HHAS10 | Bacteria | *Pseudomonadota* | *Zoogloea* sp. | N/A | Activated sludge | Fujii *et al.*, 2024 |

*1 Not listed under the ICNP.

*2 Activated sludge: strains isolated from activated sludge foams. Lab: strains used in laboratory host range analysis.

*3 Classification follows the GTDB taxonomy.

**Supplemental datasheet (Excel file)**

Datasheet summarizing the results of the analysis for comparative genomic analysis (**Fig. 2 and 5**).

**Table S4**. Basic metadata of metagenome-assembled genomes (MAGs) used in this study.

**Table S5**. Source dataset used to generate Figure 4, including processed numerical values and MAG accessions.

**Table S6**. Raw metabolic annotation output generated by DRAM.

**Table S7**. Presence/absence profiles of genes involved in peptidoglycan biosynthesis among the analyzed MAGs.

**Table S8**. Presence/absence profiles of genes related to purine biosynthesis pathways.

**Table S9**. Presence/absence profiles of genes related to pyrimidine biosynthesis pathways.

**Table S10**. Presence/absence profiles of genes associated with phospholipid biosynthesis pathways.

**Table S11**. Presence/absence profiles of genes involved in Type IV pilus assembly and biogenesis.

**Table S12**. Presence/absence profiles of genes related to flagellar assembly and biosynthesis.
